# How comprehensive is comprehensive? Prevalence of searches beyond peer-reviewed journal articles and bibliographic sources: a cross-sectional study of systematic reviews

**DOI:** 10.1017/rsm.2026.10086

**Published:** 2026-05-05

**Authors:** Jane O’Sullivan, Sarah Dawson, Chris Cooper, Julian Piers Thomas Higgins

**Affiliations:** 1 Anaesthesia and Critical Care, University Hospital Limerick, Ireland; 2 Population Health Sciences, University of Bristol Population Health Sciences, UK; 3 Population Health Sciences, University of Bristol Medical School, UK; 4 Population Health Sciences, University of Bristol, UK

**Keywords:** systematic review, information resources, bibliographic databases, evidence synthesis

## Abstract

To examine the extent to which information sources other than journal articles are sought for systematic reviews. Cross-sectional study of published systematic reviews. We examined all published systematic reviews included in MEDLINE in a 4-week period in 2019. Both systematic reviews and protocols of reviews were eligible for inclusion. (1) Number and types of information sources sought in systematic reviews; (2) proportion of reviews that explicitly searched for study reports other than journal articles; (3) proportion of reviews that searched resources containing study reports other than journal articles. A total of 1,262 systematic reviews fulfilled the eligibility criteria. The median number of information resources searched for all systematic reviews was 4. Of the 1,262 reviews, study reports other than journal articles were sought in 40% (*n* = 502) of systematic reviews (97% (*n* = 64) of Cochrane reviews and 37% (*n* = 438) of non-Cochrane reviews). Trial registers were searched in 88% of Cochrane reviews and 21% of non-Cochrane reviews. In 99.3% (*n* = 1,253) of all the systematic reviews, the searches performed had the potential to identify study reports other than journal articles. Between a third and a half of systematic reviews search for study reports other than journal articles. Systematic review searches often search resources that include study reports other than journal articles, whether or not the reviewers explicitly sought them.

## Highlights

### What is already known?

- Systematic reviewers are generally encouraged to seek information sources beyond journal articles.
- There is considerable variation in whether and how systematic reviewers search for study reports other than journal articles.

### Potential impact for RSM readers

- Around two-fifths of systematic reviews actively seek study reports other than journal articles.
- Most systematic reviewers encounter reports other than journal articles in their search.

## 1 Introduction

The validity and usefulness of a systematic review depends on it including evidence from all eligible primary research.^1^ Systematic reviews should include a comprehensive search whose objective is to identify literature related to every study addressing the research question(s).^2^ It is usually recommended that a comprehensive search goes beyond journal articles, and often that it seeks unpublished literature.^2^ ‘Published research’ refers to research findings shared with the wider academic or professional community through journals, books, or other publications for evaluation and dissemination.

The term ‘grey literature’ is often associated with non-journal articles. The concept of grey literature has been conceived as literature published outside the remit of books or journals.^3^ Further definitions exist.^4^ Grey literature is often not peer-reviewed and not indexed in the major bibliographic databases.^5^ Hence, grey literature falls beyond the remit of traditional journal articles, although grey literature is generally not considered to be synonymous with ‘non-journal’ articles. Nonetheless, a ‘grey literature search’ indicates that non-journal articles have been sought. Since ‘grey literature’ includes a very wide variety of sources, and no universally accepted definition of the term is available, we generally avoid the term in this paper, focusing instead on a more precise specification of different sources of evidence that might be considered for a systematic review.

A comprehensive search is paramount to reduce bias in a systematic review.^6^ An important bias is publication bias, which refers to the situation in which the publication status or time to publication of a study is determined by the direction or magnitude of the estimated quantities or *P* value.^6^ The bias leads to a distortion in the estimate of intervention effectiveness obtained in a meta-analysis.^7^ ^,^ ^8^ This may have serious clinical implications, for example, if a meta-analysis suffers from underreporting of adverse effects in the published studies.^9^ However, a comprehensive search that goes beyond journal articles is time-consuming and resource-intensive.^10^

A recent review underlines the importance of performing a comprehensive and extensive literature review, though points out that there is no consensus on how to perform an all-encompassing search consistently across reviews.^1^ The *Cochrane Handbook for Systematic Reviews of Interventions* recommends that ‘grey literature’ be sought where eligible and appropriate.^2^ Other guidance such as the Agency for Healthcare Research and Quality and the Centre for Reviews and Dissemination recommend searching for grey literature.^11^ Search methods for comprehensive searches have been discussed.^7^ ^,^ ^12^ ^–^ ^14^ However, no primary study has examined the prevalence of a comprehensive search for non-journal articles among systematic reviews.

We aimed to determine how often authors of systematic reviews search for study reports beyond journal articles. To achieve this, we examined a representative sample of systematic reviews to determine whether the reviews specified they performed a comprehensive search strategy, a search for specific reports other than journal articles, or a search of information resources primarily comprised of reports other than journal articles. Our aim is descriptive and classificatory: to characterise how systematic reviews report sources beyond journal articles and to present a transparent taxonomy distinguishing explicit searching from potential encounters with such literature, we do not make causal inferences about the impact of these different approaches to searching.

## 2 Methods

### 2.1 Search and selection of studies

We searched Ovid MEDLINE for systematic reviews published during a 4-week period spanning October/November 2019, following the approach used by Page et al.^15^ The search used the following terms: (systematic review or meta-analysis).pt. or (network meta-analysis.sh.) or ((cochrane database of systematic reviews or cochrane library).jn. or 1469-493X.is.) or (systematic reviews.jn. or 2046-4053.is.) or (systematic review.ti. and review.pt.) or ((meta-analys* or meta analys* or metaanalys* or meta synth* or meta-synth* or metasynth*).ti. and review.pt.) OR ((QUADAS or (diagnostic adj2 accuracy)).tw,kf,kw. and review.pt.) or ((sensitivity and specificity).sh. and review.pt.) or (prognos*.ti,hw. and review.pt.).

A single reviewer (JOS) examined the titles and abstracts of all records retrieved by the search in relation to the following inclusion criteria.

- A clear statement of criteria for including studies in the review was provided.
- An attempt at a comprehensive search for studies meeting the review’s eligibility criteria was performed, evidenced by being either stated directly or inferred from the authors’ use of two or more bibliographic databases. We used at least two bibliographic databases as a pragmatic indicator that a reasonably broad search was intended, rather than as evidence that a fully comprehensive search was undertaken.
- The search methods were documented, reporting at a minimum the information resources (e.g., bibliographic databases) examined.
- The review was reported in English.

We included reviews and protocols for reviews regardless of the nature of their question (e.g., intervention, test accuracy, prognosis, aetiology, or descriptive) and regardless of the nature of the included studies (e.g., randomised trials, other experimental studies, observational studies, qualitative research studies). Reviews were included irrespective of whether a meta-analysis was performed. We excluded reviews with a single author on the basis that they are very unlikely to be systematic (since key tasks will not have been duplicated independently). We excluded letters, comments, narrative reviews, non-systematic reviews (even if a meta-analysis or meta-synthesis was performed), summaries of systematic reviews, and systematic reviews that had been retracted or withdrawn.

We retrieved the full text of any systematic reviews appearing to meet our inclusion criteria, and a single reviewer (JOS) examined it to determine eligibility. Any discrepancies were resolved by one of the other authors.

### 2.2 Data extraction and analysis

A single reviewer (JOS) extracted information from each review onto a pre-piloted data collection form. Information was collected related to the resources searched, including bibliographic and other databases, clinical trial registries, and forwards or backwards citation searches.

We defined journal articles as full-length articles published within a scientific journal (including editorials, letters, and comments). To define more explicitly a search for study reports other than journal articles, we draw a distinction between individual literature *items* (primary source documents or other items, e.g., a specific PhD thesis) and *information resources* (bibliographic databases or other resources that might be searched to identify items, e.g., the ProQuest Dissertations & Theses Global database). Throughout the manuscript, we refer to items other than journal articles as NJAs (‘non-journal-article’ items) to avoid convoluted language. Our categorisation of *items* is provided in Appendix A of the Supplementary Material, where we also distinguish between NJAs we consider likely to have been filtered or critiqued scientifically (e.g., theses/dissertations with committee oversight, conference proceedings with abstract review, regulatory documents/clinical study reports, technical reports, and scholarly monographs) and NJAs we consider unlikely to have been filtered or critiqued scientifically (e.g., blogs, press releases, general webpages, news media, and preprints). We considered conference abstracts to be NJAs even though some are published as supplements to commercial journals.

We categorised information *resources* searched into three types. First, an information resource for journal articles (R-JA) is one whose primary content is journal articles (e.g., MEDLINE). Second, an information resource of NJAs (R-NJA) is one whose primary content is NJAs (e.g., trial registries, theses/dissertation portals, regulatory repositories, governmental/NGO report repositories, and preprint servers). Third, an information resource with mixed content (R-Mix) is one whose primary content is a combination of journal articles and other items (e.g., multidisciplinary bibliographic databases that index journal articles conference abstracts and dissertations). These first three classifications were based solely on content overviews provided by the database hosts and are listed in Appendix B of the Supplementary Material. For the purposes of this study, we interpreted the specification of seeking ‘grey literature’ in the review methods as entailing a search for NJAs.^1^ Fourth, an information resource not otherwise specified (NOS) is one when authors reported a non-journal information resource generically without naming a specific platform (e.g., ‘websites’, ‘specialist repositories’, ‘manufacturer/agency websites’), for example, stating ‘we searched websites without naming specific sites’.

We categorised each systematic review as having performed a search for NJAs if it:

1. explicitly sought items we classified as NJA items (as defined in Appendix A of the Supplementary Material); or
2. explicitly stated that it sought non-peer reviewed, grey literature, or unpublished materials (surrogates for NJAs);
3. explicitly searched an information resource we classified as a R-NJA (as defined in Appendix B of the Supplementary Material).

We further categorised each systematic review as having *potentially* encountered NJAs if it:

1. explicitly searched an information resource we classified as R-Mix (as defined in Appendix B of the Supplementary Material);
2. explicitly searched an information resource not otherwise specified (NOS)—when authors reported a resource containing NJAs generically without naming a specific platform (e.g., stating they searched ‘websites’, ‘specialist repositories’, or ‘manufacturer/agency websites’);
3. explicitly examined reference lists (backward citation searching); or
4. explicitly used forward citation searching.

The analysis was primarily descriptive with data summarised as frequencies for categorical data or average values for quantitative data.

To explore topic-related heterogeneity, we classified each included review by the primary disciplinary domain of the journal in which it was published. Each (non-Cochrane) journal could be associated with up to six codes from Elsevier’s All Sciences Journal Classification (ASJC) system.^16^ These were mapped to subject descriptions using the official taxonomy (336 unique codes). On the basis of these codes, we assigned each journal to one of seven categories: (1) Biomedical, Biostatistics, and Laboratory Sciences; (2) Clinical and Procedural Medicine; (3) Internal Medicine and Chronic Disease; (4) Mental, Neurological, and Behavioural Health; (5) Pharmacology and Therapeutics; (6) Public Health and Health Systems; (7) Reproductive, Maternal, and Child Health. The first author made a judgement regarding the journal’s predominant audience and content. Where a journal plausibly spanned multiple domains, the category that best reflected its core emphasis was selected; borderline cases were resolved by author adjudication with reference to scope notes and recent article exemplars. A complete journal-to-category mapping is provided in Appendix C of the Supplementary Material. We then undertook subgroup analyses according to these seven categories of (i) explicit searches for NJAs and (ii) potential encounters with NJAs via mixed-content sources (R-Mix).

Our objective was hypothesis generation rather than formal hypothesis testing. Accordingly, we limited analyses to descriptive summaries (counts, proportions, as appropriate) and did not perform inferential statistics, P values, or multiple-comparison adjustments.

## 3 Results

**Figure 1:**
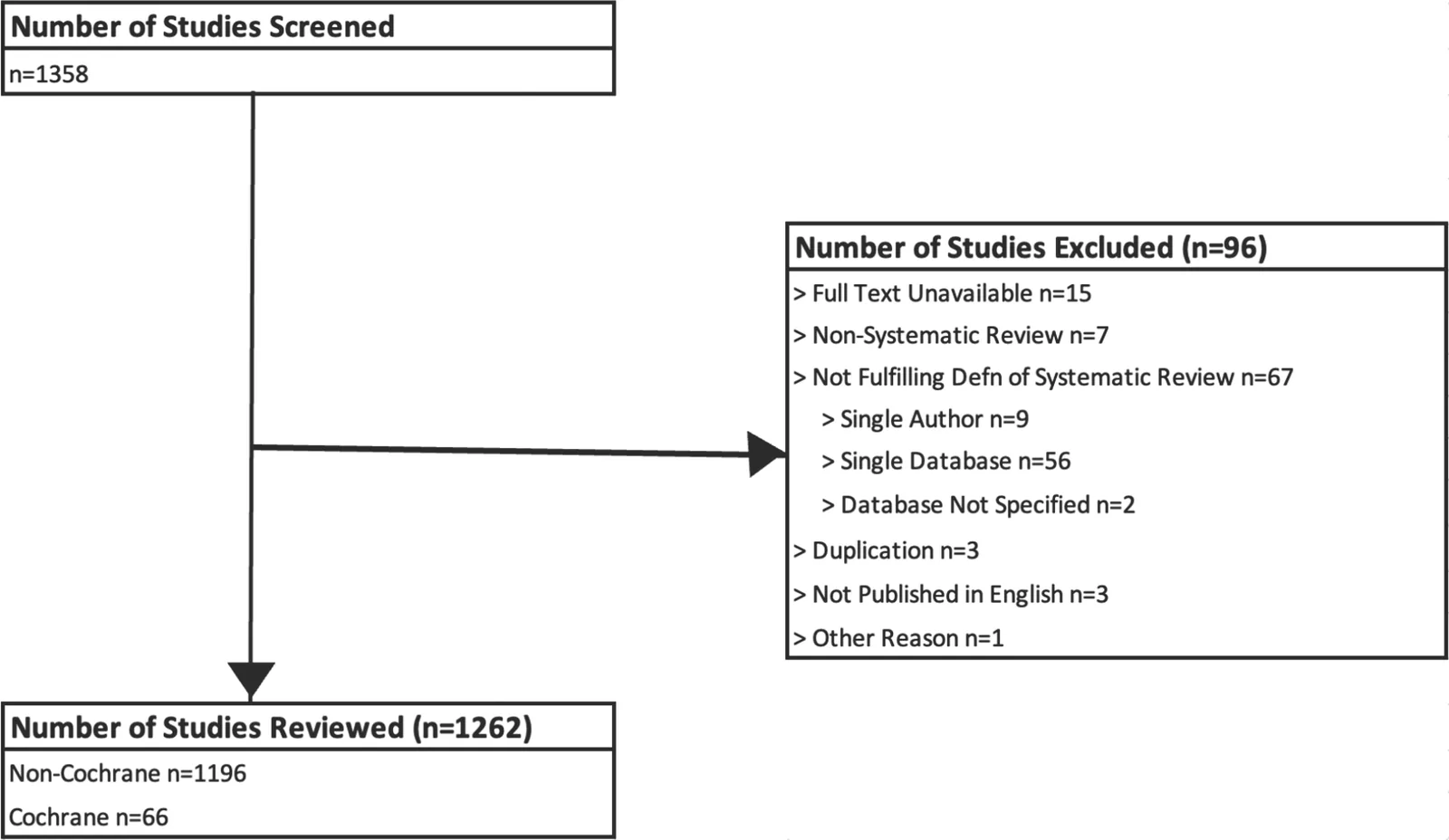
Flow chart illustrating selection of reviews into the empirical study. Figure 1 Long description.

After deduplication, our MEDLINE search identified 1,358 possible systematic reviews (see Figure 1). We were unable to access 15 of these and 67 did not fulfil our criteria for inclusion in the study. Fourteen were excluded for other reasons (see Figure 1 for further detail), leaving 1,262 systematic reviews being included in the study. Of these, 95% (*n* = 1,196) were non-Cochrane systematic reviews and 5% (*n* = 66) were Cochrane reviews (see Supplementary Data).

**Figure 2:**
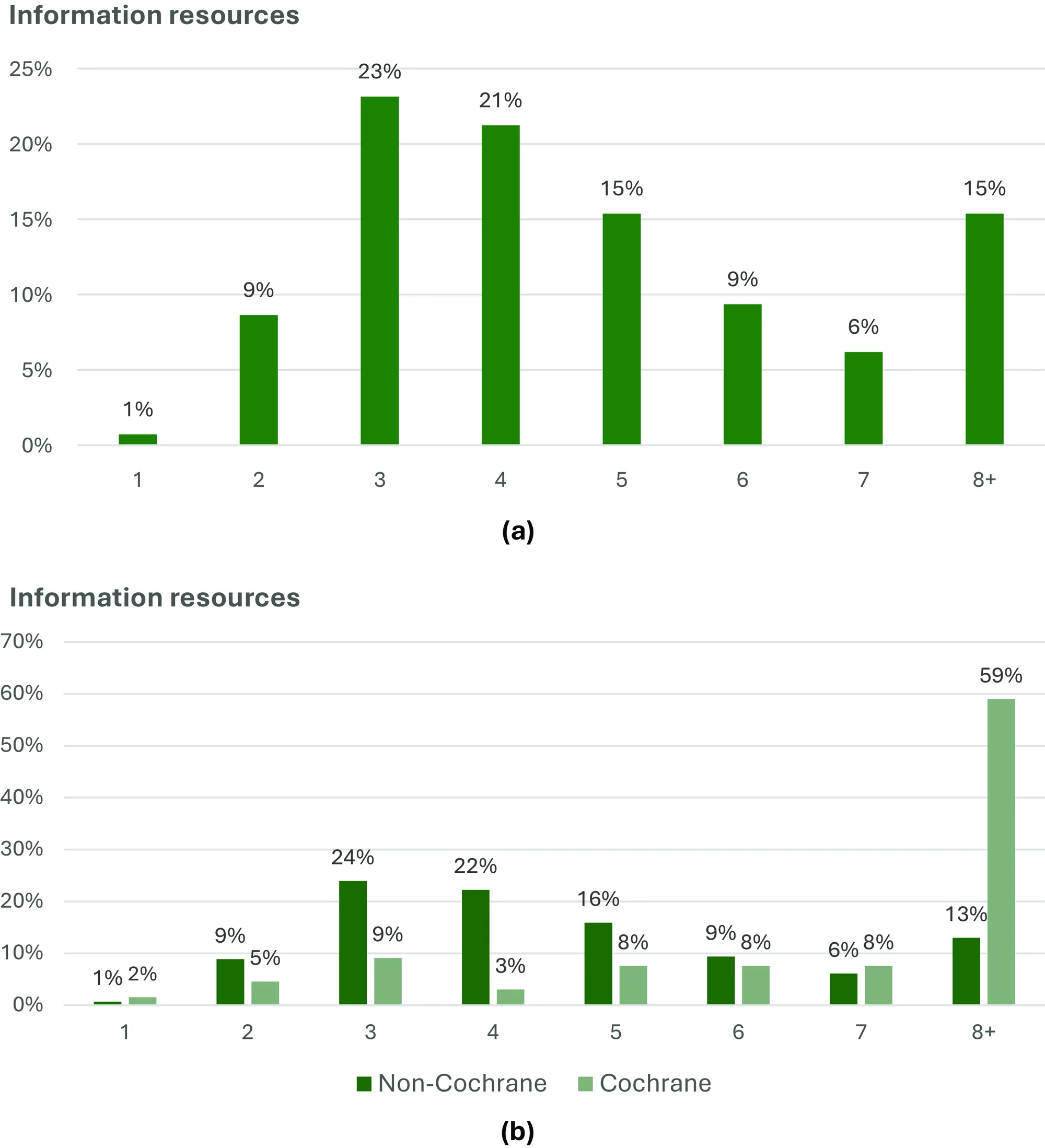
Numbers of information resources searched by the 1,262 systematic reviews included the following: (a) in total and (b) separately for Non-Cochrane and Cochrane reviews. Figure 2 Long description.

The median number of information resources searched for a systematic review was 4, with a mode of 3 (see Table 1, Figure 2). The maximum number of information resources in any systematic review was 22. Cochrane reviews generally searched more information resources, with a median of 8 compared with a median of 4 for non-Cochrane reviews (Table 1). More than half of the Cochrane reviews searched 8 or more information resources (Figure 2).

**Table 1:** Numbers of information resources searched all 1,262 systematic reviews included Table 1 Long description.

	Median	Mode	Maximum
All reviews (*n* = 1,262)	4	3	22
Non-Cochrane reviews (*n* = 1,196)	4	3	22
Cochrane reviews (*n* = 66)	8	9	22

The most common information resources of journal articles (R-JAs), resources other than journal articles (R-NJAs), and mixed resources (R-PNJAs) are listed in Table 2. PubMed and/or MEDLINE was the most common information resource for journal articles, being used in 97.6% (*n* = 1,232) of included reviews. The most common information resource potentially containing non-journal articles was Excerpta Medica Database (Embase), also used in 67% (*n* = 846) of included reviews. The most common information resource of non-journal articles (excluding trial registries) was contacting experts, being used in 6% (*n* = 78) of included reviews. The most commonly used trials registry, ClinicalTrials.gov, was searched in 14% (*n* = 173) of included reviews.

**Table 2:** The most commonly searched information resources among the 1,262 reviews included Table 2 Long description.

	Most-searched journal articles resources (R-JA)	No. of reviews	% of reviews
1	PubMed and/or MEDLINE	1,232	97.6%
2	Cumulative Index to Nursing and Allied Health Literature	254	20.1%
3	Cochrane Database of Systematic Reviews	46	3.6%
4	Chinese Biomedical Database	36	2.9%
5	VIP Chinese Science and Technology Periodicals Full Text Database	35	2.8%
	Most-searched resources of NJAs (R-NJA)	No. of reviews	% of reviews
1	ClinicalTrials.gov	173	13.7%
2	ICTRP	84	6.7%
3	Contact experts	78	6.2%
4	Search conference proceedings	68	5.4%
5	OpenGrey	52	4.1%
	Most-searched mixed resources potentially		
	including NJAs (R-Mix)	No. of reviews	% of reviews
1	Excerpta Medica Database (Embase)^a^	846	67.0%
2	Web of Science (Core Content/NOS)	485	38.4%
3	SCOPUS	319	25.3%
4	Cochrane Library	311	24.6%
5	Cochrane Central Register of Controlled Trials	265	21.0%

a Embase indexes many conference abstracts.

Of the total 1,262 systematic reviews, 19% (*n* = 245) reported that they explicitly sought NJAs or grey literature, 11% (*n* = 138) that they explicitly searched for items we classified as NJA items, and 20% (*n* = 251) that they explicitly searched information resources we classified as R-NJAs (see Appendix B of the Supplementary Material). A small proportion (6%) of the 1,262 reviews explicitly reported that they excluded ‘grey literature’ (*n* = 76).

**Figure 3:**
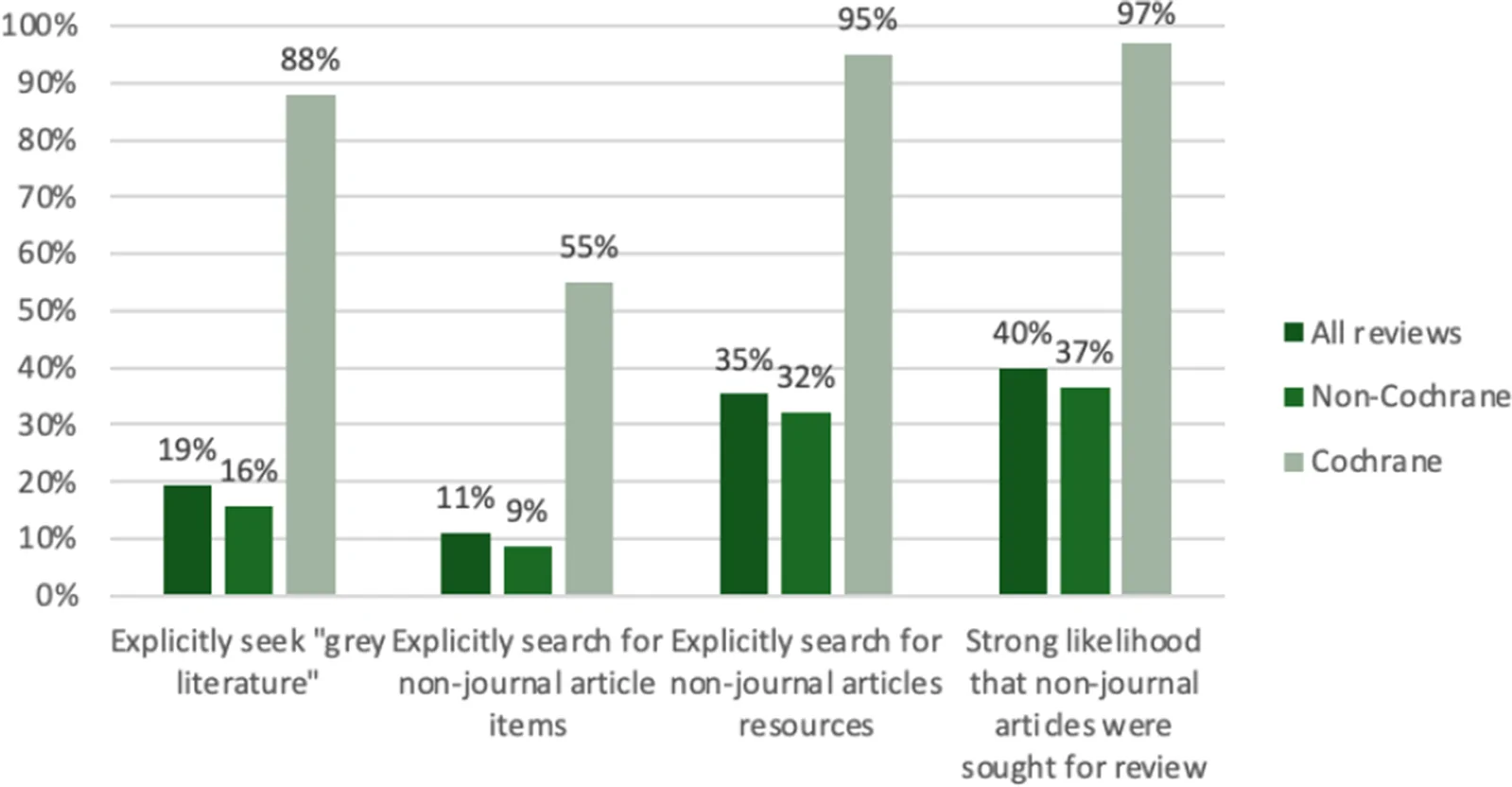
Proportions of reviews considered to have performed a search for reports other than journal articles, for all reviews (*n* = 1,262), non-Cochrane reviews (*n* = 1,196), and Cochrane reviews (*n* = 66). Figure 3 Long description.

Overall, we judged that 40% (*n* = 502) of the 1,262 reviews included a search beyond journal articles (Figure 3). This was much higher for Cochrane reviews (97%, *n* = 64) than for non-Cochrane reviews (37%, *n* = 438) (Figure 3).

**Figure 4:**
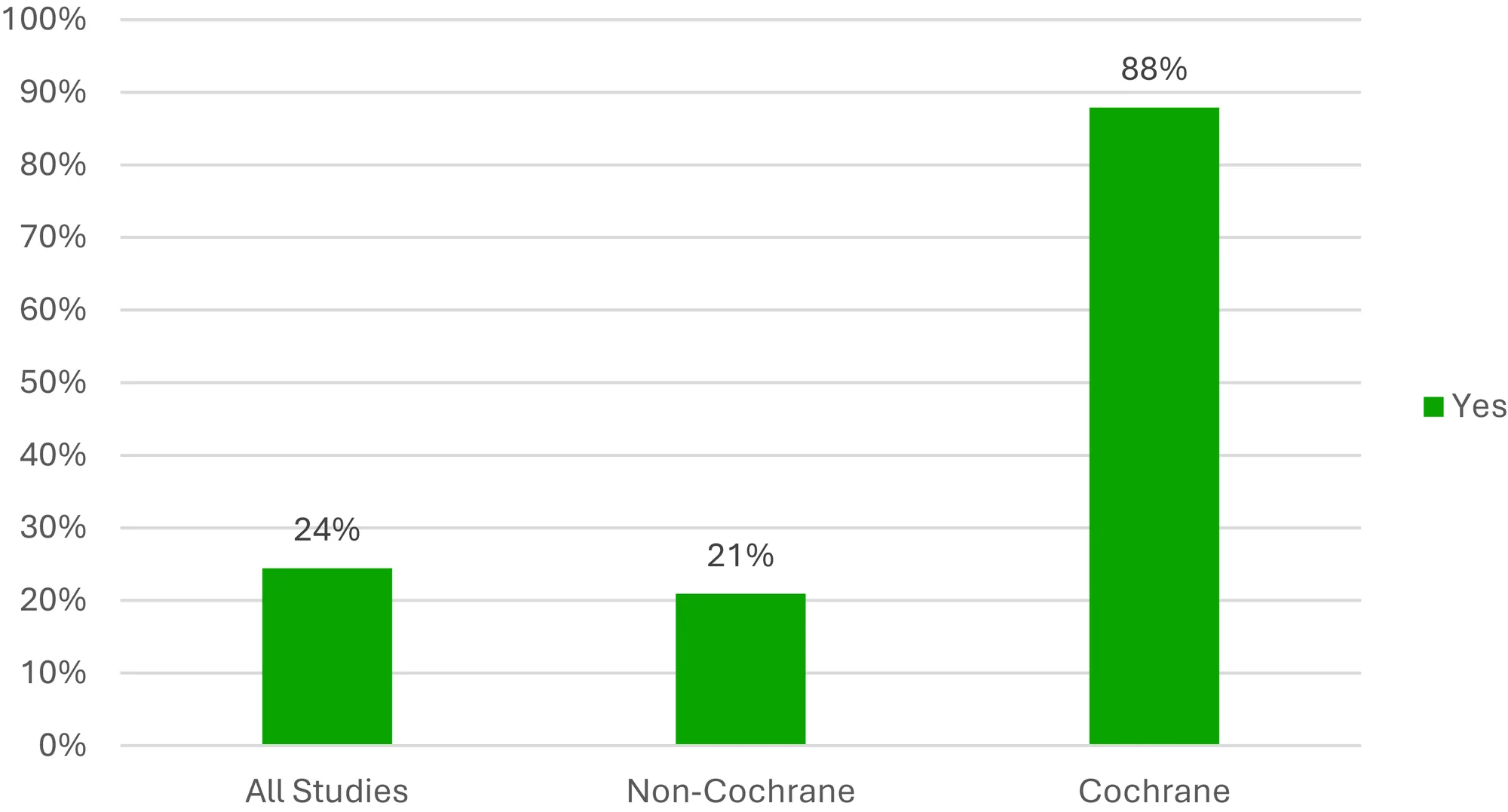
Proportions of reviews explicitly searching trials registries.

We observed that 24% (*n* = 308) of the reviews searched trials registries, including most of the Cochrane reviews (Figure 4). Separately, 40% (*n* = 502) explicitly searched for NJAs overall. A considerably higher proportion of Cochrane reviews than non-Cochrane reviews sought these; we judged that 97% (*n* = 64) of Cochrane reviews and 37% (*n* = 438) of non-Cochrane reviews potentially regarded NJAs as eligible *and* performed a search for NJAs.

**Figure 5:**
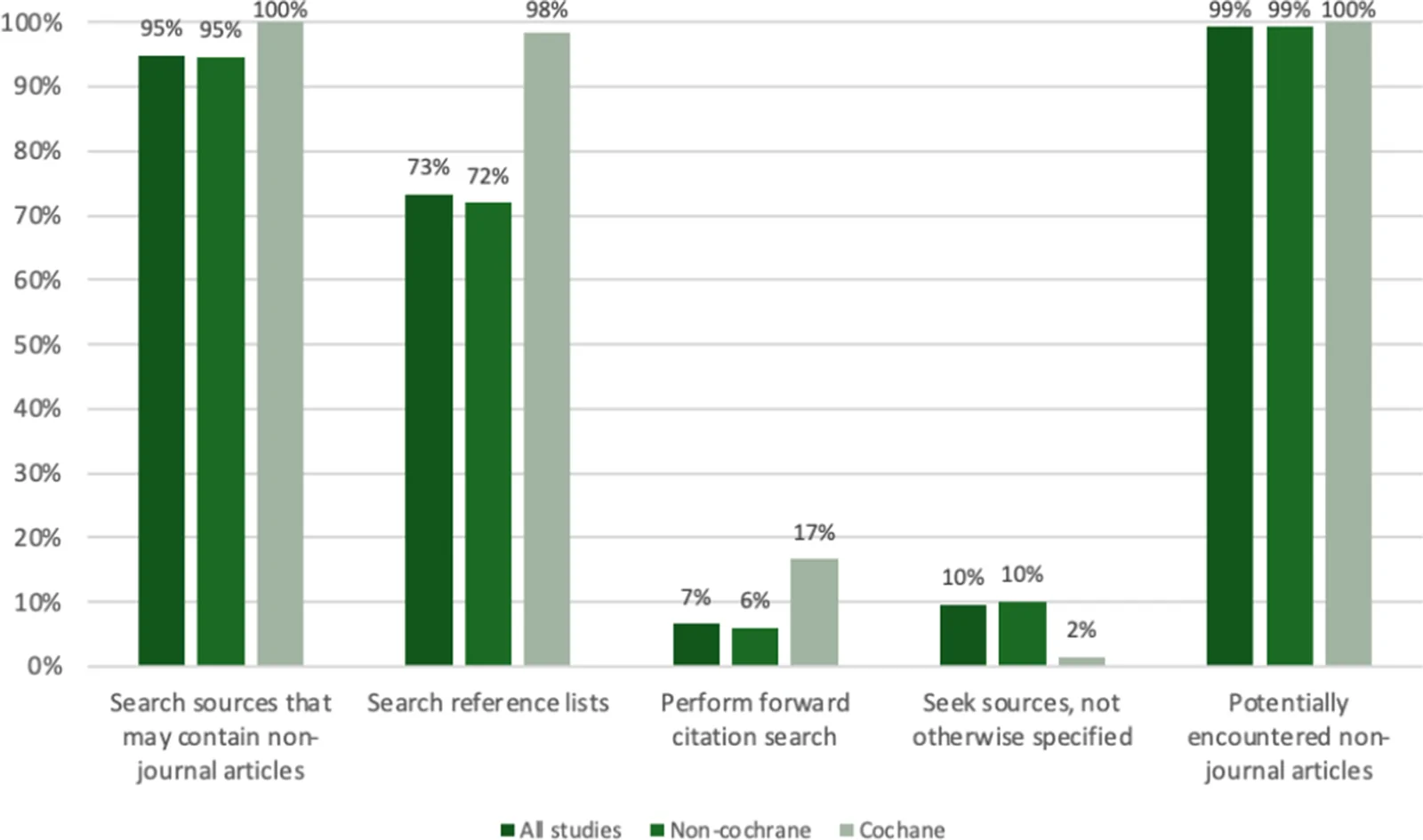
Proportions of reviews that potentially encountered NJAs, for all reviews (*n* = 1,262), non-Cochrane reviews (*n* = 1,196), and Cochrane reviews (*n* = 66). Figure 5 Long description.

Our categorisation of whether reviews potentially encountered NJAs led to 99.3% (*n* = 1,253) of 1,262 reviews being considered to have done this (Figure 5). The proportions for non-Cochrane reviews and Cochrane reviews were 99.2% (*n* = 1,186) and 100% (*n* = 66), respectively.

In our classification of the journals publishing each of the individual reviews (*n* = 1,196), the largest groups were Clinical and Procedural Medicine at 27% (*n* = 317) and Internal Medicine and Chronic Disease at 23% (*n* = 267). Mid-sized categories were Biomedical, Biostatistics, and Laboratory Sciences at 14% (*n* = 165), Public Health and Health Systems at 12% (*n* = 158), and Mental, Neurological, and Behavioural Health at 14% (*n* = 142). Reproductive, Maternal, and Child Health accounted for 7% (*n* = 86) and Pharmacology and Therapeutics accounted for 5% (*n* = 61). (Percentages do not sum to 100% due to rounding.) Results of the subgroup analyses are provided in Supplementary Figure 1. Reviews in Public Health and Health Systems and Reproductive, Maternal, and Child Health typically showed the strongest orientation towards non-journal sources, whereas fewer reviews in Biomedical, Statistics and Laboratory Sciences sought NJAs.

## 4 Discussion

Our cross-sectional study of 1,262 systematic reviews contained in MEDLINE over a 4-week period found that less than half (40%) of systematic reviews performed an explicit search for reports other than journal articles (NJAs). However, over 99% of the systematic reviews had the potential to encounter study reports other than journal articles through the information resources they searched. This large difference in proportions reflects the problem that systematic review authors were often ambiguous about whether they explicitly searched NJAs, which may reflect suboptimal reporting and a lack of awareness of what was held in the information resources.

Previous studies have examined the related question of searching for unpublished studies, focussing on narrower concepts than our consideration of NJAs. Hartling et al.^17^ assessed 129 systematic reviews from three Cochrane review groups *(Acute Respiratory Infections, Infectious Diseases, and Developmental Psychosocial and Learning Problems*). They found that 96.1% of reviews searched for unpublished studies, primarily by assessing the reference list of unpublished research items. The study was restricted to Cochrane reviews that included a meta-analysis and presented effect sizes, which may have led to the inclusion of reviews of higher methodological quality. Additionally, they assessed the methods to determine if the authors specifically sought grey literature or dissertations, although they did not account for search methods looking for obvious grey literature sources or items beyond dissertations.

Korevaar et al. sought to determine the number of information resources of unpublished data used by diagnostic systematic reviews.^18^ They included 100 non-Cochrane and 100 Cochrane diagnostic reviews. They observed that 76% and 98% of non-Cochrane and Cochrane reviews, respectively, searched beyond bibliographic databases. Corresponding figures from our study are 37% and 97% in non-Cochrane and Cochrane studies, respectively. Korevaar et al found that 22% and 68% of non-Cochrane and Cochrane reviews looked in databases specifically for grey literature, including trial registries. These findings contrast with ours (37% and 97%). This difference may be explained by a much higher proportion of trial registries cited by the reviews we included in our study.

To our knowledge, this is the largest study to have investigated how often NJAs have been sought or encountered for inclusion in systematic reviews. Our sample included all types of systematic reviews, regardless of their classification. We specified a novel set of criteria to determine whether systematic reviewers had undertaken an explicit search for NJAs, recognising that a search for NJAs can mean either an explicit search for them (e.g., searching ‘grey literature’) or searching information resources that contain them. We acknowledge that systematic reviewers may potentially have encountered NJAs through the resources they search without being clear about whether they are actively seeking them (e.g., using reference lists, forward searching, or searching information resources that are not clearly specified), and we analysed explicit searches for NJAs and searches potentially retrieving NJAs separately.

Sub-category patterns in our study differed across health care domains. Reviews in Public Health and Health Systems more frequently reported consulting NJAs, suggesting a greater practical reliance on such sources in some public health-oriented domains, consistent with prior observations in public health evidence synthesis^4^ rather than evidence of greater impact on summary effect estimates. By contrast, Biomedical, Statistics, and Laboratory Sciences rely most heavily on peer-reviewed experimental literature, so non-journal searching was comparatively rarer. We observed that Cochrane reviews were in general much more likely to search for, and potentially to encounter, NJAs. In future research, it would be interesting to examine the extent to which this finding persists within topic areas.

Our study nonetheless has limitations. First, we searched a single 4-week period of systematic reviews indexed in MEDLINE, so would not have included reviews in journals not indexed by this database. Second, approximately 10% of reviews searched an information resource without precisely specifying what the resource was. Most of these were categorised as potentially encountering NJAs, with only three being considered to have searched for NJAs explicitly. Third, our assessments were based only on the search methods described in the systematic reviews; we did not look at what types of reports were actually assessed by the review authors. Fourth, we classified the information resource based solely on the content overview provided by the creators. While this was a consistent and reproducible approach, we are aware that not all database descriptions fully characterise the database contents. For example, the description of CINAHL does not indicate that articles other than journal articles are included, leading to its classification as a journal article resource, whereas we are aware that it contains items we would classify as NJAs. Finally, we did not separate between protocols and completed reviews. Further research could usefully examine whether search plans are implemented as intended. Further research should also address the question of whether the inclusion of NJAs impacts on the sets of studies included in systematic reviews and/or on the conclusions of the reviews.

The term ‘grey literature’ has several different meanings in the literature. Moreover, there appears to be fluidity in understanding of what constitutes grey literature and ‘white’ or ‘conventional’ literature.^19^ Rather than focus on whether or not a grey literature search was conducted, we focused on the identification of study reports other than journal articles. We believe this enables a novice reviewer to understand better the concept of a comprehensive search strategy and to make informed decisions on what types of sources to search for. We developed and implemented a broad set of criteria to classify literature searches in relation to NJAs, based on defining individual study reports as journal articles or NJAs. We suggest that our classification scheme may have broader application in the understanding of search strategies and systematic review searches and might form the basis of refined guidance on when and where to search these sources in future systematic reviews.

## Supporting information

10.1017/rsm.2026.10086.sm001 O’Sullivan et al. supplementary material 1 O’Sullivan et al. supplementary material

10.1017/rsm.2026.10086.sm002 O’Sullivan et al. supplementary material 2 O’Sullivan et al. supplementary material

## Data Availability

Data availability is not applicable to this article as no new data were created or analysed in this study.

## Supplementary material

To view supplementary material for this article, please visit http://doi.org/10.1017/rsm.2026.10086.

## Long descriptions

### Figure 1 Long description

The flowchart consists of three main boxes connected by arrows.

* At the top is a box titled Number of Studies Screened with n equals 1358.

* A vertical arrow points down from the top box to the bottom box. Halfway down this arrow, a horizontal arrow branches off to the right.

* The right-hand box is titled Number of Studies Excluded (n equals 96). It lists the following reasons for exclusion:

* Full Text Unavailable n equals 15.

* Non-Systematic Review n equals 7.

* Not Fulfilling Defn of Systematic Review n equals 67. This is further broken down into Single Author n equals 9, Single Database n equals 56, and Database Not Specified n equals 2.

* Duplication n equals 3.

* Not Published in English n equals 3.

* Other Reason n equals 1.

* The bottom box, where the vertical arrow terminates, is titled Number of Studies Reviewed (n equals 1262). It is divided into two categories:

* Non-Cochrane n equals 1196.

* Cochrane n equals 66.

Navigate back to Figure 1.

### Table 1 Long description

The table consists of four columns: Review Category, Median, Mode, and Maximum.

Row 1: All reviews (n = 1,262) shows a Median of 4, a Mode of 3, and a Maximum of 22.

Row 2: Non-Cochrane reviews (n = 1,196) shows a Median of 4, a Mode of 3, and a Maximum of 22.

Row 3: Cochrane reviews (n = 66) shows a Median of 8, a Mode of 9, and a Maximum of 22.

Navigate back to Table 1.

### Figure 2 Long description

A multi-panel figure with two vertical bar charts labeled a and b. Both charts share an x-axis titled Information resources with categories 1, 2, 3, 4, 5, 6, 7, and 8 plus.

Panel a shows the total percentage of reviews. The y-axis ranges from 0 percent to 25 percent. The data peaks at 3 resources with 23 percent and 4 resources with 21 percent. Other values are 1 resource at 1 percent, 2 at 9 percent, 5 at 15 percent, 6 at 9 percent, 7 at 6 percent, and 8 plus at 15 percent.

Panel b compares Non-Cochrane reviews in dark green and Cochrane reviews in light gray. The y-axis ranges from 0 percent to 70 percent.

* For 1 resource: Non-Cochrane 1 percent, Cochrane 2 percent.

* For 2 resources: Non-Cochrane 9 percent, Cochrane 5 percent.

* For 3 resources: Non-Cochrane 24 percent, Cochrane 9 percent.

* For 4 resources: Non-Cochrane 22 percent, Cochrane 3 percent.

* For 5 resources: Non-Cochrane 16 percent, Cochrane 8 percent.

* For 6 resources: Non-Cochrane 9 percent, Cochrane 8 percent.

* For 7 resources: Non-Cochrane 6 percent, Cochrane 8 percent.

* For 8 plus resources: Non-Cochrane 13 percent, Cochrane 59 percent.

Navigate back to Figure 2.

### Table 2 Long description

The table is divided into three sections.

Section 1: Most-searched journal articles resources (R-J A).

1. PubMed and or MEDLINE: 1,232 reviews (97.6 percent).

2. Cumulative Index to Nursing and Allied Health Literature: 254 reviews (20.1 percent).

3. Cochrane Database of Systematic Reviews: 46 reviews (3.6 percent).

4. Chinese Biomedical Database: 36 reviews (2.9 percent).

5. V I P Chinese Science and Technology Periodicals Full Text Database: 35 reviews (2.8 percent).

Section 2: Most-searched resources of N J A s (R-N J A).

1. ClinicalTrials.gov: 173 reviews (13.7 percent).

2. I C T R P: 84 reviews (6.7 percent).

3. Contact experts: 78 reviews (6.2 percent).

4. Search conference proceedings: 68 reviews (5.4 percent).

5. OpenGrey: 52 reviews (4.1 percent).

Section 3: Most-searched mixed resources potentially including N J A s (R-Mix).

1. Excerpta Medica Database (Embase): 846 reviews (67.0 percent).

2. Web of Science (Core Content or N O S): 485 reviews (38.4 percent).

3. SCOPUS: 319 reviews (25.3 percent).

4. Cochrane Library: 311 reviews (24.6 percent).

5. Cochrane Central Register of Controlled Trials: 265 reviews (21.0 percent).

Navigate back to Table 2.

### Figure 3 Long description

The Y-axis represents percentages from 0 percent to 100 percent in increments of 10 percent. The X-axis contains four categories of search behavior. A legend on the right identifies three groups: All reviews (dark green), Non-Cochrane (medium green), and Cochrane (light grey).

* Explicitly seek grey literature: All reviews 19 percent, Non-Cochrane 16 percent, Cochrane 88 percent.

* Explicitly search for non-journal article items: All reviews 11 percent, Non-Cochrane 9 percent, Cochrane 55 percent.

* Explicitly search for non-journal articles resources: All reviews 35 percent, Non-Cochrane 32 percent, Cochrane 95 percent.

* Strong likelihood that non-journal articles were sought for review: All reviews 40 percent, Non-Cochrane 37 percent, Cochrane 97 percent.

Across all categories, Cochrane reviews show significantly higher proportions of searching for non-journal reports compared to non-Cochrane reviews.

Navigate back to Figure 3.

### Figure 5 Long description

The Y-axis represents percentages from 0 percent to 100 percent in increments of 10 percent. The X-axis lists five categories. A legend at the bottom identifies three bars for each category: All studies (dark green), Non-cochrane (medium green), and Cochrane (light gray).

* Search sources that may contain non-journal articles: All studies 95 percent, Non-cochrane 95 percent, Cochrane 100 percent.

* Search reference lists: All studies 73 percent, Non-cochrane 72 percent, Cochrane 98 percent.

* Perform forward citation search: All studies 7 percent, Non-cochrane 6 percent, Cochrane 17 percent.

* Seek sources, not otherwise specified: All studies 10 percent, Non-cochrane 10 percent, Cochrane 2 percent.

* Potentially encountered non-journal articles: All studies 99 percent, Non-cochrane 99 percent, Cochrane 100 percent.

Navigate back to Figure 5.
