# Supplementary material for: How comprehensive is comprehensive? Prevalence of searches beyond peer-reviewed journal articles and bibliographic sources: a cross-sectional study of systematic reviews
Source: Res Synth Methods. 2026 May 5;17(6):1210–20. doi: 10.1017/rsm.2026.10086 (PMC13732357; doi:10.1017/rsm.2026.10086)
Supplement: Supplementary file 1 [file S1759287926100866sup001.docx]

**Supplementary materials**

**Appendix A:** **Classification of literature items as journal articles (JAs) and reports other than journal articles (NJAs)**

| *JAs and NJAs likely to be scientifically critiqued (NJA(C))* | | *NJAs unlikely to be scientifically critiqued (NJA(U))* | |
| --- | --- | --- | --- |
| **Research report materials** | | **Manuscript (unpublished)** | |
| Journal article | JA | Pre-print | NJA(U) |
| Journal editorial | JA | **Statistical materials** | |
| Letter to editor | JA | Excel spreadsheet | NJA(U) |
| Comment/reply (published) | JA | **Miscellaneous** | |
| Erratum (published) | JA | Hearing | NJA(U) |
| **Academic institution material** | | Interview | NJA(U) |
| Thesis or dissertation | NJA(C) | Unpublished materials (otherwise unspecified) | NJA(U) |
| Monograph | NJA(C) | **General public information materials** | |
| **Library catalogue materials** | | Blog | NJA(U) |
| Book | NJA(C) | Discussion forum | NJA(U) |
| Book section | NJA(C) | Film or broadcast | NJA(U) |
| Catalogue | NJA(C) | Magazine article | NJA(U) |
| Electronic book | NJA(C) | Newspaper article | NJA(U) |
| Electronic book section | NJA(C) | Online multimedia | NJA(U) |
| Encyclopaedia | NJA(C) | Pamphlet | NJA(U) |
| Electronic encyclopaedia | NJA(C) | Podcast | NJA(U) |
| **Scientific meeting materials** | | Press release | NJA(U) |
| Conference abstract | NJA(C) | Social media | NJA(U) |
| **Expert communications/ & materials** | | Television | NJA(U) |
| Expert consensus (document) | NJA(C) | Web page | NJA(U) |
| **Instructional material** | | **Scientific meeting materials** | |
| Guideline | NJA(C) | Conference keynote speech | NJA(U) |
| Standard | NJA(C) | Conference poster | NJA(U) |
| Drug label | NJA(C) | Conference - other | NJA(U) |
| Handbook | NJA(C) | PowerPoint presentation | NJA(U) |
| Instrument (e.g. BDI) | NJA(C) | **Expert communications/ & materials** | |
| Safety sheet | NJA(C) | Personal communication | NJA(U) |
| **Legal & regulatory documents** | |  |  |
| Bill | NJA(C) |  |  |
| Government document | NJA(C) |  |  |
| Legal rule or regulation | NJA(C) |  |  |
| Regulatory statute | NJA(C) |  |  |
| Green paper | NJA(C) |  |  |
| Letter (regulatory approval) | NJA(C) |  |  |
| Licensing approval document | NJA(C) |  |  |
| Patent | NJA(C) |  |  |
| **Manuscript (unpublished)** | |  |  |
| Clinical study report | NJA(C) |  |  |
| **Statistical materials** | |  |  |
| Research dataset | NJA(C) |  |  |
| Online raw data database | NJA(C) |  |  |
| **Clinical trial register** | |  |  |
| Registry record | NJA(C) |  |  |
| **Miscellaneous** | |  |  |
| Grant | NJA(C) |  |  |

**Appendix B:** **Classification of information resources as primarily containing journal articles (R-JA), primarily containing sources other than journal articles (R-NJA), or resources containing both journal articles and sources other than journal articles (R-Mix)**

| **Bibliographic or journal database*** | **Acronym** | **Also known as** | **Subject** | **Region** | **Type** | | **Primary sources** | | **Our classification** | **Referenced† (n=)** |  |  |
| --- | --- | --- | --- | --- | --- | --- | --- | --- | --- | --- | --- | --- |
| Africa-Wide Information |  |  | Multidisciplinary | Region-specific | Bibliographic database | | Journals, books, reports, theses, etc | | R-MIX | 3 |  |  |
| ** Africa Journals Online Library* | AJOL |  | Multidisciplinary | Region-specific | Bibliographic database | | Journals | | R-JA | 3 |  |  |
| AgEcon Search |  |  | Subject-specific | Global | Bibliographic database | | Journals, conference presentations, working papers, government documents, theses and dissertations | | R-MIX | 1 |  |  |
| Agricola |  |  | Subject-specific | Global | Bibliographic database | | Journals, monographs, theses, patents, multimedia, reports | | R-MIX | 1 |  |  |
| Airiti Library |  |  | Multidisciplinary | Region-specific | Bibliographic database | | Journals, theses, conference proceedings, e-books, periodicals | | R-MIX | 2 |  |  |
| AllConferences.com |  |  | Multidisciplinary | Global | Bibliographic database | | Conference Abstracts | | **R-NJA** | 2 |  |  |
| American Society of Clinical Oncology | ASCO |  | Multidisciplinary | Region-specific | Bibliographic database | | Conference proceedings | | **R-NJA** | 2 |  |  |
| Anthropology Plus |  |  | Subject-specific | Global | Bibliographic database | | Journals, reports, commentaries, edited works and obituaries | | R-MIX | 2 |  |  |
| APA Psych Articles |  |  | Subject-specific | Region-specific | Bibliographic database | | Journals | | R-JA | 12 |  |  |
| APA Psych Extra |  |  | Subject-specific | Region-specific | Bibliographic database | | Journals, conference proceedings, newsletters, reports, monographs, standards, guidelines, patient information | | R-MIX | 1 |  |  |
| ArticleFirst |  |  | Multidisciplinary | Global | Bibliographic database | | Journals | | R-JA | 1 |  |  |
| arXiv |  |  | Multidisciplinary | Global | Bibliographic database | | Pre-prints | | **R-NJA** | 1 |  |  |
| Arzteblatt |  |  | Multidisciplinary | Region-specific | Bibliographic database | | Journals, conferences | | R-MIX | 1 |  |  |
| Association for Computing and Machinery Digital Library | ACM |  | Subject Specific | Global | Bibliographic database | | Journals, conference proceedings, technical magazines, newsletters, books | | R-MIX | 4 |  |  |
| AuSport Med |  |  | Subject-specific | Region-specific | Bibliographic database | | Journals | | R-JA | 1 |  |  |
| Bavarian State Library | BSB | Bayerische Staatsbibliothek | Multidisciplinary | Region-specific | Bibliographic database | | Journals, Books, Magazines, Newspapers | | R-MIX | 1 |  |  |
| BioMed Central | BMC |  | Multidisciplinary | Global | Bibliographic database | | Journals | | R-JA | 2 |  |  |
| bioRxiv |  |  | Multidisciplinary | Global | Bibliographic database | | Pre-prints | | **R-NJA** | 1 |  |  |
| British Education Index |  |  | Subject-specific | Global | Bibliographic database | | Journals, theses | | R-MIX | 2 |  |  |
| British Nursing Index | BNI |  | Subject-specific | Global | Bibliographic database | | Journals | | R-JA | 1 |  |  |
| CancerLit |  |  | Subject-specific | Global | Bibliographic database | | Journals, government reports, conference proceedings, monographs | | R-MIX | 2 |  |  |
| CAPES Periodic | CAPES | Coordenacao de Aperfeicoamento de Pessoal de Nivel Superior | Multidisciplinary | Region-specific | Bibliographic database | | Journals, references, patents, statistics, audiovisual materials, theses, dissertations, books, reference works | | R-MIX | 4 |  |  |
| CAPES Thesis bank |  | Catalogo de Teses e Dissertacoes | Multidisciplinary | Region-specific | Bibliographic database | | Theses | | **R-NJA** | 2 |  |  |
| Center for International Rehabilitation Information and Exchange | CIRRIE | International Rehabilitation Information | Subject-specific | Region-specific | Bibliographic database | | Journals, books, reports, organisational data | | R-MIX | 1 |  |  |
| Chicano Database |  |  | Subject Specific | Region-specific | Bibliographic database | | Journals, newspapers, books, magazines | | R-MIX | 1 |  |  |
| ChinaInfo | INTBIT | China InfoBank | Subject-specific | Region-specific | Bibliographic database | | Journals, news, announcements, reports, government documents | | R-MIX | 1 |  |  |
| ChinaResource |  | European Center for Digital Resources in Chinese Studies (ChinaResource.org) | Multidisciplinary | Region-specific | Bibliographic database | | Journals and books | | R-MIX | 1 |  |  |
| Chinese Biomedical Database | CBM | Chinese Biomedical Literature Database | Multidisciplinary | Region-specific | Bibliographic database | | Journals | | R-JA | 36 |  |  |
| Chinese Network Knowledge Infrastructure | CNKI |  | Multidisciplinary | Region-specific | Bibliographic database | | Journals, Newspapers, Theses, Conference proceedings | | R-MIX | 95 |  |  |
| ** China Academic Journals* | CAJ | China Academic Journals Full Text | Multidisciplinary | Region-specific | Bibliographic database | | Journals | | R-JA | 4 |  |  |
| ** China Core Newspapers* | CCND |  | Multidisciplinary | Region-specific | Bibliographic database | | Newspapers | | **R-NJA** | 0 |  |  |
| ** China Doctors/Masters Dissertations* | CCMD |  | Multidisciplinary | Region-specific | Bibliographic database | | Theses | | **R-NJA** | 0 |  |  |
| ** China Proceedings of Conferences* | CPCD |  | Multidisciplinary | Region-specific | Bibliographic database | | Conference proceedings | | **R-NJA** | 0 |  |  |
| ClinicaKey |  |  | Multidisciplinary | Global | Bibliographic database | | Journals, EBM, books, multimedia, guidelines | | R-MIX | 2 |  |  |
| Cochrane Library |  |  | Multidisciplinary | Global | Bibliographic database | | Journals (Systematic Reviews), Journals, conference proceedings, EBM compilations, regulatory data, trial | | R-MIX | 311 |  |  |
| ** Cochrane Central Register of Controlled Trials* | CENTRAL |  | Multidisciplinary | Global | Bibliographic database | | Journals, conference proceedings, EBM compilations, regulatory data, trial register data | | R-MIX | 265 |  |  |
| ** Cochrane Clinical Answers* | CCA |  | Multidisciplinary | Global | EBM Database | | EBM Compilation | | R-MIX | 0 |  |  |
| ** Cochrane Database of Systematic Reviews* | CDSR |  | Multidisciplinary | Global | Bibliographic database | | Journals (Systematic Reviews) | | R-JA | 46 |  |  |
| Cochrane (not otherwise specified) |  | Cochrane Collaboration/CRS Web | Multidisciplinary | Global | Bibliographic database | | Journals (Systematic Reviews), Journals, conference proceedings, EBM compilations, regulatory data, trial | | R-R-MIX | 98 |  |  |
| Community Care Inform Adults |  |  | Subject-specific | Global | Bibliographic database | | Practice guidance, legal information and educational tools | | **R-NJA** | 1 |  |  |
| Conference Papers Index |  |  | Multidisciplinary | Global | Bibliographic database | | Conference proceedings | | **R-NJA** | 1 |  |  |
| Cost Effectiveness Analysis Registry | CEA REGISTRY | Tufts Cost Effectiveness Analysis Registry | Subject-specific | Global | Bibliographic database | | Cost-effectiveness analyses summaries | | **R-NJA** | 4 |  |  |
| Current Chemical Reactions Database | CCR | Current Contents | Subject-specific | Global | Bibliographic database | | Journals | | R-JA | 1 |  |  |
| Current Contents Connect |  |  | Multidisciplinary | Global | Bibliographic database | | Journals | | R-JA | 1 |  |  |
| DailyMed |  |  | Subject-specific | Global | Bibliographic database | | FDA Records | | **R-NJA** | 1 |  |  |
| Dialnet |  |  | Multidisciplinary | Region-specific | Bibliographic database | | Journals, monographs, theses, conference proceedings, magazines | | R-MIX | 1 |  |  |
| Digital Access to Research Theses | DART | Digital Access to Research Theses (DART)‐Europe E‐theses Portal | Multidisciplinary | Region-specific | Bibliographic database | | Theses | | **R-NJA** | 1 |  |  |
| Directory of Open Access Journal | DOAJ |  | Multidisciplinary | Global | Bibliographic database | | Journals | | R-JA | 2 |  |  |
| Disaster Information Management Research Center | DIMRC |  | Subject-specific | Global | Bibliographic database | | Journals, conferences | | R-MIX | 1 |  |  |
| EBSCO (not otherwise specified) |  | EBSCO Host | Multidisciplinary | Global | Bibliographic database | | Journals, books, conference proceedings, magazines | | R-MIX | 23 |  |  |
| ** Academic Search Complete* |  | Academic Search Ultimate | Multidisciplinary | Global | Indexing and Abstracts | | Journals | | R-JA | 7 |  |  |
| ** AgeLine* |  |  | Subject-specific | Global | Bibliographic database | | Journals, books, book chapters, reports | | R-MIX | 1 |  |  |
| ** Allied and Complementary Medicine Database* | AMED |  | Subject-specific | Global | Bibliographic database | | Journals, conferences | | R-MIX | 24 |  |  |
| ** AltHealth Watch* |  |  | Subject-specific | Global | Bibliographic database | | Journals | | R-JA | 1 |  |  |
| ** Biological Abstracts* |  |  | Multidisciplinary | Global | Bibliographic database | | Journals | | R-A | 1 |  |  |
| ** Biosis Previews* |  |  | Multidisciplinary | Global | Bibliographic database | | Journals, books, conference proceedings, patents | | R-MIX | 12 |  |  |
| ** Business Source Complete* |  | Business Source Ultimate | Subject-specific | Global | Bibliographic database | | Journals | | R-JA | 1 |  |  |
| ** CAB Abstracts* |  |  | Multidisciplinary | Global | Bibliographic database | | Journals, conference proceedings, reports | | R-MIX | 3 |  |  |
| ** Criminal Justice Abstracts* |  |  | Subject-specific | Global | Bibliographic database | | Journals | | R-JA | 1 |  |  |
| ** Cumulative Index to Nursing and Allied Health Literature* | CINAHL |  | Subject-specific | Global | Bibliographic database | | Journals | | R-JA | 254 |  |  |
| ** Dentistry and Oral Science Source* | DOSS |  | Subject-specific | Global | Bibliographic database | | Journals | | R-JA | 1 |  |  |
| ** EBSCOHost eBook Collection* |  |  | Multidisciplinary | Global | Bibliographic database | | Books | | R-MIX | 1 |  |  |
| ** EconLit* |  |  | Subject-specific | Global | Bibliographic database | | Journals, books, conference proceedings, theses | | R-MIX | 7 |  |  |
| ** Education Resources Information Centre* | ERIC |  | Subject-specific | Global | Bibliographic database | | Journals, books, conference proceedings, guidelines, theses, regulatory documents | | R-MIX | 25 |  |  |
| ** Education Source* |  |  | Subject-specific | Global | Bibliographic database | | Journals, conference proceedings, multimedia materials | | R-MIX | 2 |  |  |
| ** Global Health* |  |  | Subject-specific | Global | Bibliographic database | | Journals, serials, books, reports, conference proceedings, patents, theses, | | R-MIX | 13 |  |  |
| ** Greenfile* |  |  | Subject-specific | Global | Bibliographic database | | Journals, governmental documents | | R-MIX | 1 |  |  |
| ** Health Business* |  | Health Source: Nursing /Academic Edition | Subject-specific | Global | Bibliographic database | | Journals | | R-JA | 1 |  |  |
| ** Health Source* |  |  | Subject-specific | Global | Bibliographic database | | Journals | | R-JA | 1 |  |  |
| ** International Database for Medical Literature* |  | Medical Literature Analysis and Retrieval System Online | Multidisciplinary | Global | Bibliographic database | | Journals | | R-JA | 0 |  |  |
| ** Music Index* |  |  | Subject-specific | Global | Bibliographic database | | Journals | | R-JA | 1 |  |  |
| ** Nursing Reference Center Plus* |  |  | Subject-specific | Global | Bibliographic database | | Journals, books, EBM bulletins, | | R-MIX | 1 |  |  |
| ** Psychology and Behavioral Sciences Collection* |  |  | Subject-specific | Global | Bibliographic database | | Journals | | R-JA | 3 |  |  |
| ** PSYNDEX* |  |  | Subject-specific | Region-specific | Bibliographic database | | Journals, monographs, reports | | R-MIX | 4 |  |  |
| ** SocIndex* |  |  | Subject-specific | Global | Bibliographic database | | Journals | | R-JA | 1 |  |  |
| ** SPORTDiscus* |  |  | Subject-specific | Global | Bibliographic database | | Journals, books, dissertations, | | R-MIX | 37 |  |  |
| EHRAF World Cultures |  |  | Subject-specific | Global | Bibliographic database | | Journals and books | | R-MIX | 1 |  |  |
| EMCare |  |  | Subject-specific | Global | Bibliographic database | | Journals | | R-JA | 2 |  |  |
| EnfermeíaA(F)isioterapia-Podología | ENFISPO |  | Subject-specific | Region-specific | Bibliographic database | | Journals | | R-JA | 1 |  |  |
| Europe PubMed Central |  |  | Multidisciplinary | Region-specific | Bibliographic database | | Journals, pre-prints, micropublications, books, reviews, protocols | | R-MIX | 1 |  |  |
| Evidence for Policy and Practice Information and Coordinating Centre | EPPI-CENTRE |  | Multidisciplinary | Global | Bibliographic database | | Systematic Reviews and Regulatory Documents | | R-MIX | 2 |  |  |
| Excerpta Medica database | EMBASE |  | Multidisciplinary | Global | Bibliographic database | | Journals, conference proceedings, regulatory information, EBM compilations | | R-MIX | 846 |  |  |
| F1000 Research |  |  | Multidisciplinary | Global | Bibliographic database | | Journals, Protocols, Opinions, Editorials | | R-MIX | 1 |  |  |
| Food Science and Technology Abstracts | FSTA |  | Subject-specific | Global | Bibliographic database | | Journals | | R-JA | 1 |  |  |
| Gale Academic One File |  |  | Multidisciplinary | Global | Bibliographic database | | Journals | | R-JA | 1 |  |  |
| GEOBASE |  |  | Subject-specific | Global | Bibliographic database | | Journals, conference proceedings, chapters | | R-MIX | 1 |  |  |
| Grey Literature Database |  | Medicine Gray Literature Repor/ GreyLit/New York Academy of Medicine Grey Literature Report | Multidisciplinary | Global | Bibliographic database | | Grey Literature (not otherwise specified) | | **R-NJA** | 1 |  |  |
| Health Economic Evaluation Database | HEED |  | Subject-specific | Global | Bibliographic database | | Journals, economic evaluations | | R-MIX | 1 |  |  |
| Health Evidence |  |  | Multidisciplinary | Global | Bibliographic database | | Systematic Reviews | | R-MIX | 2 |  |  |
| Health Management Information Consortium | HMIC |  | Subject-specific | Global | Bibliographic database | | Journals and grey literature (not otherwise specified) | | R-MIX | 5 |  |  |
| Health Services Research Projects in Progress | HSR PROJ |  | Multidisciplinary | Global | Bibliographic database | | Dataset | | **R-NJA** | 1 |  |  |
| Health Systems Evidence | HSE |  | Multidisciplinary | Region-specific | Bibliographic database | | Evidence bulletins, systematic reviews, economic evaluationns | | R-MIX | 1 |  |  |
| Healthcare Database Advanced Search | HDAS |  | Multidisciplinary | Global | Bibliographic database | | Journals, conference proceedings, grey literature (not otherwise specified) | | R-MIX | 1 |  |  |
| HealthSTAR |  |  | Multidisciplinary | Global | Bibliographic database | | Journals | | R-JA | 2 |  |  |
| Highwire Press |  |  | Multidisciplinary | Global | Bibliographic database | | Journals, pre-prints, books, multimedia | | R-MIX | 1 |  |  |
| Hinari |  |  | Multidisciplinary | Global | Bibliographic database | | Journals and books | | R-MIX | 1 |  |  |
| HSELINE Database |  |  | Subject-specific | Global | Bibliographic database | | Journals, books, reports | | R-MIX | 1 |  |  |
| IC-SMOKE Database |  |  | Subject-specific | Global | Bibliographic database | | Systematic Reviews | | R-MIX | 1 |  |  |
| IndMed |  |  | Multidisciplinary | Region-specific | Bibliographic database | | Journals | | R-JA | 3 |  |  |
| Informit (Health Collection) |  |  | Multidisciplinary | Region-specific | Bibliographic database | | Journals, Books, Conference proceedings | | R-NJA | 2 |  |  |
| Institute for Clinical and Economic Review | ICER |  | Subject-specific | Global | Bibliographic database | | Reports | | **R-NJA** | 2 |  |  |
| Institute of Electronic and Electrical Engineers Xplore | IEEE XPLORE |  | Subject-specific | Global | Bibliographic database | | Journals, conference proceedings, technical standards, books | | R-MIX | 4 |  |  |
| Institutional/University Database |  |  | Multidisciplinary | Region-specific | Bibliographic database | | (not otherwise specified) | | R-MIX | 8 |  |  |
| International Association for Dental Research and its regional subgroups | IADR |  | Subject-specific | Global | Bibliographic database | | Journals | | R-JA | 1 |  |  |
| International Bibliography of Social Science | IBSS |  | Subject-specific | Global | Bibliographic database | | Journals and books | | R-MIX | 1 |  |  |
| International Federation of Pharmaceutical Manufacturers and Associations | IFPMA |  | Subject-specific | Global | Bibliographic database | | Pharmaceutical Reports | | R-MIX | 1 |  |  |
| International Guidelines Network | G-I-N |  | Multidisciplinary | Global | Bibliographic database | | Guidelines | | **R-NJA** | 3 |  |  |
| International Occupational Safety and Health Information Centre Database | CISDOC |  | Subject-specific | Global | Bibliographic database | | Journals, legal standards, data sheets, training materials, book, standards | | R-MIX | 1 |  |  |
| International Pharmaceutical Abstracts |  |  | Subject-specific |  | Bibliographic database | |  | | R-JA | 1 |  |  |
| International Scientific Indexing | ISI |  | Multidisciplinary | Global | Bibliographic database | | Journals | | R-JA | 1 |  |  |
| Iran MEDEX |  |  | Multidisciplinary | Region-specific | Bibliographic database | | Journals | | R-JA | 2 |  |  |
| Iranian Research Institute for Information Science and Technology | IRANDOC |  | Multidisciplinary | Region-specific | Bibliographic database | | Journals, technical reports, dissertations, conference proceedings, technical papers | | R-MIX | 5 |  |  |
| ISC Research |  |  | Subject-specific | Region-specific | Bibliographic database | | Dataset | | **R-NJA** | 1 |  |  |
| J-Stage |  | Igaku-Chuo-Zasshi/Ichushi-Web | Multidisciplinary | Region-specific | Bibliographic database | | Journals, conference proceedings, reports | | R-MIX | 4 |  |  |
| Joanna Briggs Institute Database of Systematic Reviews and Implementation Reports | JBI |  | Multidisciplinary | Region-specific | Bibliographic database | | Journals | | R-MIX | 2 |  |  |
| Joanna Briggs Institute (not otherwise specified) | JBI |  | Multidisciplinary | Region-specific | Bibliographic database | | Journals, EBM Bulletins | | R-MIX | 1 |  |  |
| Journals@Ovid | OVFT |  | Multidisciplinary | Global | Bibliographic database | | Journals, books, editorials | | R-MIX | 2 |  |  |
| KoreaMed |  |  | Multidisciplinary | Region-specific | Bibliographic database | | Journals | | R-JA | 2 |  |  |
| Korean Institute for Science and Technology Information | KISTI |  | Multidisciplinary | Region-specific | Bibliographic database | | Journals, theses, guidelines | | R-MIX | 1 |  |  |
| Korean Medical Database | KMBASE |  | Multidisciplinary | Region-specific | Bibliographic database | | Journals | | R-JA | 2 |  |  |
| Korean studies information service system | KISS |  | Multidisciplinary | Region-specific | Bibliographic database | | Journals, books | | R-MIX | 1 |  |  |
| MagIran |  |  | Multidisciplinary | Region-specific | Bibliographic database | | Journals | | R-JA | 11 |  |  |
| Manual, Alternative and Natural Therapy Index System Database | MANTIS |  | Subject-specific | Global | Bibliographic database | | Journals | | R-JA | 1 |  |  |
| Maternity and Infant Care Database | MIC |  | Subject-specific | Global | Bibliographic database | | Journals, books, grey literature (not otherwise specified) | | R-MIX | 2 |  |  |
| MathSciNet |  |  | Subject-specific | Global | Bibliographic database | | Journals | | R-JA | 1 |  |  |
| McMaster PLUS |  |  | Subject-specific | Global | Bibliographic database | | Journals, guidelines, and evidence-based bulletins | | R-MIX | 1 |  |  |
| Medicine and Liberty | MEDLIB |  | Multidisciplinary | Global | Bibliographic database | | Educational Materials | | **R-NJA** | 1 |  |  |
| Mendeley |  |  | Multidisciplinary | Global | Bibliographic database | | Dataset | | **R-NJA** | 1 |  |  |
| National Criminal Justice System |  |  | Subject-specific | Global | Bibliographic database | | Federal, state and local government reports, books, journals, research reports, unpublished materials | | R-MIX | 1 |  |  |
| National Digital Library of Theses and Dissertations | NDLTD | Digital Dissertations | Multidisciplinary | Global | Bibliographic database | | Theses | | **R-NJA** | 2 |  |  |
| National Guideline Clearing House | AHRQ |  | Multidisciplinary | Global | Bibliographic database | | Guidelines | | **R-NJA** | 4 |  |  |
| National Information Center on Health Services Research and Health Care Technology | NICHSR |  | Subject-specific | Global | Bibliographic database | | Journals | | R-JA | 1 |  |  |
| National Institute for Occupational Safety and Health | NIOSH | NIOSHTIC‐2 | Subject-specific | Global | Bibliographic database | | Journals, conference proceedings, bulletins, technical reports, EBM solutions | | R-MIX | 3 |  |  |
| National Library Medicine | NCBI |  | Multidisciplinary | Global | Bibliographic database | | Journals | | R-JA | 2 |  |  |
| ** NLM Catalogue and Bookshelf* |  |  | Multidisciplinary | Global | Bibliographic database | | Journals, books, audiovisuals, other materials (not otherwise specified) | | R-MIX | 1 |  |  |
| ** PubMed and/or MEDLINE* |  |  | Multidisciplinary | Global | Bibliographic database | | Journals | | R-JA | 1232 |  |  |
| ** Toxicology Information Online* | TOXLINE |  | Subject-specific | Global | Bibliographic database | | Journals | | R-JA | 1 |  |  |
| National Research Register |  |  | Multidisciplinary | Region-specific | Bibliographic database | | Journals and books | | R-MIX | 1 |  |  |
| National Technical Information Service | NTIS |  | Multidisciplinary | Global | Bibliographic database | | Journals, conference proceedings, theses, government documents, reports, books | | R-MIX | 1 |  |  |
| National Transportation Library | NTL Resources |  | Subject-specific | Global | Bibliographic database | | Journals, reports, datasets | | R-MIX | 1 |  |  |
| NeuroBITE | PsychBITE |  | Subject-specific | Global | Bibliographic database | | Journals, books, conference proceedings, bibliographic lists | | R-MIX | 1 |  |  |
| New York Academy of Medicine Library Online Catalog |  |  | Multidisciplinary | Global | Bibliographic database | | Journals, books, grey literature (not otherwise specified) | | R-MIX | 2 |  |  |
| Occupational Therapy Systematic Evaluation of Evidence | OTSeeker |  | Subject-specific | Global | Bibliographic database | | Journals, conference proceedings, textbooks, experts | | R-MIX | 6 |  |  |
| Open Access Theses and Dissertations | OATD |  | Multidisciplinary | Global | Bibliographic database | | Theses | | **R-NJA** | 4 |  |  |
| Open Directory of Open Access Repositories |  |  | Multidisciplinary | Global | Bibliographic database | | Journals, theses, conference proceedings, reports, datasets, patents | | R-MIX | 1 |  |  |
| OpenGrey |  |  | Multidisciplinary | Global | Bibliographic database | | Research reports, theses, conference proceedings, grey literature (not otherwise specified) | | **R-NJA** | 52 |  |  |
| Open System for Information on Grey Literature in Europe | SIGLE |  | Multidisciplinary | Global | Bibliographic database | | Grey Literature (not otherwise specified) | | **R-NJA** | 10 |  |  |
| OpenThesis |  |  | Multidisciplinary | Global | Bibliographic database | | Theses | | **R-NJA** | 1 |  |  |
| Ovid Northern Light Life Sciences Conference Abstracts |  |  | Multidisciplinary | Global | Bibliographic database | | Conference proceedings | | **R-NJA** | 1 |  |  |
| Ovid Nursing Database |  |  | Subject-specific | Global | Bibliographic database | | Journals and books | | R-MIX | 1 |  |  |
| Physical Education Index |  |  | Subject-specific | Global | Bibliographic database | | Journals, reports, conference proceedings, magazines, patents | | R-MIX | 1 |  |  |
| Physiotherapy Evidence Database | PEDRo |  | Subject-specific | Global | Bibliographic database | | Journals, systematic reviews | | R-MIX | 34 |  |  |
| POPLine |  |  | Subject-specific | Global | Bibliographic database | | Journals, theses, books, reports, conference proceedings, newspaper articles, regulatory documents | | R-MIX | 6 |  |  |
| ProQuest (not otherwise specified) |  |  | Multidisciplinary | Global | Bibliographic database | | Journals, dissertations, working papers, reports | | R-MIX | 44 |  |  |
| ** Applied Social Sciences Index and Abstracts* | ASSIA |  | Subject-specific | Global | Bibliographic database | | Journals | | R-JA | 7 |  |  |
| ** ProQuest Dissertations and Theses* |  |  | Multidisciplinary | Global | Bibliographic database | | Theses | | **R-NJA** | 18 |  |  |
| ** ProQuest Social Sciences* |  |  | Subject-specific | Global | Bibliographic database | | Journals | | R-JA | 1 |  |  |
| ** PTSDpubs* | PILOTS | The Published International Literature on Traumatic Stress | Subject-specific | Global | Bibliographic database | | Journals, books, theses, government documents | | R-MIX | 1 |  |  |
| Psicodoc |  |  | Subject-specific | Region-specific | Bibliographic database | | Journals, conference proceedings, books | | R-MIX | 1 |  |  |
| PsychInfo |  |  | Subject-specific | Global | Bibliographic database | | Journals, books, theses, conference proceedings | | R-MIX | 223 |  |  |
| PubPsych |  |  | Subject-specific | Region-specific | Bibliographic database | | Journals, Books, Conference proceedings | | R-MIX | 1 |  |  |
| Redalyc Scientific Information System | Reladlyc |  | Multidisciplinary | Region-specific | Bibliographic database | | Journals | | R-JA | 1 |  |  |
| Reed Tech Navigator™ for Drug Labels | PDR3D |  | Multidisciplinary | Region-specific | Bibliographic database | | Regulatory Data | | **R-NJA** | 1 |  |  |
| REHABDATA Database | NARIC |  | Subject-specific | Global | Bibliographic database | | Journals, reports, books | | R-MIX | 1 |  |  |
| RISS International | RISS |  | Subject-specific | Region-specific | Bibliographic database | | Journals, theses, books, serials | | R-MIX | 1 |  |  |
| SafetyLit |  |  | Subject-specific | Global | Bibliographic database | | Journals | | R-JA | 1 |  |  |
| Saudi Digital Library |  |  | Multidisciplinary | Region-specific | Bibliographic database | | Journals, books, theses, reports | | R-MIX | 1 |  |  |
| Science Information Database | SID |  | Multidisciplinary | Region-specific | Bibliographic database | | Journals | | R-JA | 11 |  |  |
| ScienceDirect |  | Elsevier | Multidisciplinary | Global | Bibliographic database | | Journals and books | | R-MIX | 67 |  |  |
| Scientific Electronic Library Online | SciELO | Electronic Scientific Library | Multidisciplinary | Region-specific | Bibliographic database | | Journals and conference proceedings | | R-MIX | 21 |  |  |
| SCOPUS |  |  | Multidisciplinary | Global | Bibliographic database | | Journals, books, conference proceedings | | R-MIX | 319 |  |  |
| SemanticsScholar |  |  | Multidisciplinary | Global | Bibliographic database | | Journals | | R-JA | 1 |  |  |
| Shodhgana |  |  | Multidisciplinary | Region-specific | Bibliographic database | | Theses | | **R-NJA** | 1 |  |  |
| SinoMed Database |  |  | Multidisciplinary | Region-specific | Bibliographic database | | Journals, theses | | R-MIX | 8 |  |  |
| Social Care Online |  | Electronic Library of Social Care | Subject-specific | Global | Bibliographic database | | Journals, legislation, government documents, guidance, systematic reviews, briefs, grey literature (not otherwise specified) and reports | | R-MIX | 1 |  |  |
| Social Science Research Network | SSRN |  | Subject-specific | Global | Bibliographic database | | Journals, conference proceedings | | R-MIX | 1 |  |  |
| Sociological Abstracts |  |  | Subject-specific | Global | Bibliographic database | | Journals, Books, Conference proceedings, theses, working papers | | R-MIX | 6 |  |  |
| ** Social Services Abstracts* |  |  | Subject-specific | Global | Bibliographic database | | Journals, Books, Conference proceedings | | R-MIX | 1 |  |  |
| Springer Link |  | Springer | Multidisciplinary | Global | Bibliographic database | | Journals, books, series, protocols, reference works and conference proceedings | | R-MIX | 7 |  |  |
| SURF Repository | SURF |  | Multidisciplinary | Global | Bibliographic database | | Dataset | | **R-NJA** | 2 |  |  |
| SveMed+ |  |  | Multidisciplinary | Region-specific | Bibliographic database | | Journals, conference proceedings,editorials, letters | | R-MIX | 1 |  |  |
| Taylor & Francis |  |  | Multidisciplinary | Global | Bibliographic database | | Journals | | R-JA | 1 |  |  |
| The British Library |  |  | Multidisciplinary | Global | Bibliographic database | | Journals, books, theses, patents, digital materials | | R-MIX | 1 |  |  |
| ** Electronic Theses Online System (EThOS)* |  |  | Multidisciplinary | Region-specific | Bibliographic database | | Theses | | **R-NJA** | 3 |  |  |
| The Chinese Medicine Database | CM-DB |  | Multidisciplinary | Region-specific | Bibliographic database | | Journals, Books | | R-MIX | 2 |  |  |
| The Conference Board | TED | Total Economy Database | Subject-specific | Global | Bibliographic database | | Economic Data | | **R-NJA** | 1 |  |  |
| The International Network of Agencies for Health Technology Assessment | INAHTRA |  | Multidisciplinary | Global | Bibliographic database | | HTAs | | **R-NJA** | 1 |  |  |
| The Networked Digital Library of Theses and Dissertations | NDLTD | Network Digital Library | Multidisciplinary | Global | Bibliographic database | | Theses | | **R-NJA** | 5 |  |  |
| The PAIS Index |  |  | Multidisciplinary | Global | Bibliographic database | | Journals, books, government reports, reports, conference proceedings, web content, grey literature (not otherwise specified) | | R-MIX | 1 |  |  |
| The reCAL Legacy |  | reCAL: The bibliographic database for prosthetics and orthotics | Subject-specific | Global | Bibliographic database | | Journals | | R-JA | 1 |  |  |
| Transfusion Evidence Library |  |  | Subject-specific | Global | Bibliographic database | | Systematic reviews, journals, conference proceedings | | R-MIX | 2 |  |  |
| TROVE National Library of Australia |  |  | Multidisciplinary | Region-specific | Bibliographic database | | Journals, books, newspapers | | R-MIX | 3 |  |  |
| VIP Chinese Science and Technology Periodicals Full Text Database | CQVIP | VIP Information/Chinese Scientific Journals Database/Chongqing VIP/Weipu | Multidisciplinary | Region-specific | Bibliographic database | | Journals | | R-JA | 35 |  |  |
| Virtual Health Library | VHL | Portal Regional da BVS | Multidisciplinary | Region-specific | Bibliographic database | | Journals, theses, monographs, books, conference proceedings, reports, government publications and publications by international organisations | | R-MIX | 12 |  |  |
| ** Latin American and Caribbean Health Sciences Literature* | LILACS (AMRO/PAHO) | Biblioteca Regional de Medicina (BIREME – Regional Library of Medicine) | Multidisciplinary | Region-specific | Bibliographic database | | Clinical Studies, Synthesis/Revisions/Guidelines, Technical Reports/Government Publications, Thesis and Dissertions | | R-MIX | 68 |  |  |
| ** Brazilian Bibliography of Dentistry* | BBO |  | Subject-specific | Region-specific | Bibliographic database | | Journals and books | | R-MIX | 4 |  |  |
| ** Carribean Health Sciences Literature* | MEDCARIB |  | Multidisciplinary | Region-specific | Bibliographic database | | Journals, books, theses, technical reports, conference proceedings | | R-MIX | 0 |  |  |
| ** Disaster Documentation Center Collection* | DESASTRES |  | Subject Specific | Global | Bibliographic database | | Journals, bibliographies, books, technical reports, conference proceedings, theses, | | R-MIX | 0 |  |  |
| ** Formative Second Opinion* |  |  | Multidisciplinary | Global | EBM Bulletin | | EBM compilation | | R-MIX | 0 |  |  |
| ** Health Information Locator* |  |  | Multidisciplinary | Global | Bibliographic database | | Health sites, portals, social networks (grey materials) | | **R-NJA** | 0 |  |  |
| ** Homeoindex* |  |  | Subject-specific | Global | Bibliographic database | | Journals | | R-JA | 0 |  |  |
| ** Medical Literature Analysis and Retrieval System Online* | MEDLARS | MEDical Literature Analysis and Retrieval System (MEDLARS) | Multidisciplinary | Global | Bibliographic database | | Journals | | R-MIX | 1 |  |  |
| ** Sanitary Engineering and Environmental Sciences* | REPIDISCA |  | Subject-specific | Global | Bibliographic database | | Journals, technical reports, theses, conference proceedings, standards, videos, software, training materials | | R-MIX | 0 |  |  |
| ** SidSaúde* |  |  | Subject-specific | Region-specific | Bibliographic database | | Journals and books | | R-MIX | 0 |  |  |
| ** Spanish Bibliographic Index on Health Sciences* | IBECS | Índice Bibliográfico Español en Ciencias de la Salud (Spanish Bibliographic Index on Health Sciences) | Multidisciplinary | Global | Bibliographic database | | Journals | | R-JA | 3 |  |  |
| ** WHOLIS* |  |  | Subject-specific | Global | Bibliographic database | | Journals, technical and political documents, organisational documents | | R-MIX | 0 |  |  |
| Wanfang Data |  |  | Multidisciplinary | Region-specific | Bibliographic database | | Journals, theses, conference proceedings | | R-MIX | 64 |  |  |
| ** Academic Conferences In China* |  |  | Multidisciplinary | Region-specific | Bibliographic database | | Conference proceedings | | **R-NJA** | 0 |  |  |
| ** China Online Journals* |  |  | Multidisciplinary | Region-specific | Bibliographic database | | Journals | | R-JA | 0 |  |  |
| ** Dissertations of China* |  |  | Multidisciplinary | Region-specific | Bibliographic database | | Theses | | **R-NJA** | 0 |  |  |
| Web of Science Core Collection | WoS | Web of Knowledge | Multidisciplinary | Global | Bibliographic database | | Journals, books, conference proceedings | | R-MIX | 14 |  |  |
| Web of Science (not otherwise specified) | WoS | Web of Knowledge | Multidisciplinary | Global | Bibliographic database | | Journals, books, conference proceedings | | R-MIX | 471 |  |  |
| ** Arts and Humanities Citation Index* | A&HCI |  | Subject-specific | Global | Bibliographic database | | Journals | | R-JA | 2 |  |  |
| ** Biosis Citation Index* |  |  | Multidisciplinary | Global | Bibliographic database | | Journals, monographs, conference proceedings, patents | | R-MIX | 0 |  |  |
| ** Book Citation Index* |  |  | Multidisciplinary | Global | Bibliographic database | | Books | | **R-NJA** | 1 |  |  |
| Conference Proceedings Citation Index | CPCI |  | Multidisciplinary | Global | Bibliographic database | | Conference proceedings | | **R-NJA** | 19 |  |  |
| ** Emerging Sources Citation Index* |  |  | Multidisciplinary | Global | Bibliographic database | | Journals | | R-JA | 3 |  |  |
| ** Science Citation Index Expanded* | SCI Expanded | Science Citation Index | Multidisciplinary | Global | Bibliographic database | | Journals | | R-JA | 24 |  |  |
| ** Social Science Citation Index* | SSCI |  | Subject-specific | Global | Bibliographic database | | Journals | | R-JA | 13 |  |  |
| What Works in Reentry Clearinghouse |  | The Clearing House | Subject-specific | Global | Bibliographic database | | Journals and reports | | R-MIX | 4 |  |  |
| WHO Global Index Medicus | GIM | WHO Global Health Library | Multidisciplinary | Global | Bibliographic database | | Journals, theses, newspapers, reports | | R-MIX | 4 |  |  |
| ** African Index Medicus* | AIM (/AFRO) |  | Multidisciplinary | Region-specific | Bibliographic database | | Journal articles, technical reports, theses and dissertations | | R-MIX | 3 |  |  |
| ** Index Medicus for the Eastern Mediterranean Region* | IMEMR (/EMRO) |  | Multidisciplinary | Region-specific | Bibliographic database | | Journals | | R-JA | 1 |  |  |
| ** Index Medicus for the South-East Asia Region* | IMSEAR (/SEARO) |  | Multidisciplinary | Region-specific | Bibliographic database | | Journals, health reports and documents | | R-MIX | 1 |  |  |
| ** Latin American and Caribbean Health Sciences Literature* | LILACS (AMRO/ PAHO) | Biblioteca Regional de Medicina (BIREME – Regional Library of Medicine) | Multidisciplinary | Region-specific | Bibliographic database | | Clinical studies, synthesis/revisions/guidelines, technical reports/government publications, thesis and dissertions | | R-MIX | 68 |  |  |
| Wiley Library Online |  |  | Multidisciplinary | Global | Bibliographic database | | Journals, books | | R-MIX | 6 |  |  |
| Worldcat |  |  | Multidisciplinary | Global | Bibliographic database | | Journals, books, theses | | R-MIX | 3 |  |  |
| ** World CAT Dissertations and Theses* |  |  | Multidisciplinary | Global | Bibliographic database | | Theses | | **R-NJA** | 3 |  |  |
| WorldSciNet |  |  | Multidisciplinary | Global | Bibliographic database | | Journals and books | | R-MIX | 1 |  |  |
| ZETOC |  |  | Multidisciplinary | Region-specific | Bibliographic database | | Journals and conference proceedings | | R-MIX | 3 |  |  |
| **Trials registers** |  |  |  |  |  | |  | |  |  |  |  |
| Australian New Zealand Clinical Trials Registry | ANZCTR |  | Multidisciplinary | Region-specific | Trials register | | Clinical trials register | | **R-NJA** | 5 |  |  |
| Chinese Clinical trials register | Chi-CTR |  | Multidisciplinary | Region-specific | Trials register | | Clinical trials register | | **R-NJA** | 3 |  |  |
| Clinical Trials |  | ClinicalTrials.gov | Multidisciplinary | Region-specific | Trials register | | Clinical trials register | | **R-NJA** | 173 |  |  |
| Clinical Trials Registry India | CTRI |  | Multidisciplinary | Region-specific | Trials register | | Clinical trials register | | **R-NJA** | 2 |  |  |
| European Union Clinical trials register | EUCTR |  | Multidisciplinary | Region-specific | Trials register | | Clinical trials register | | **R-NJA** | 8 |  |  |
| German Clinical trials register | DRKS | Deutches Register Klinischer Studien | Multidisciplinary | Region-specific | Trials register | | Clinical trials register | | **R-NJA** | 2 |  |  |
| International Clinical Trials Registry Platform | ICTRP | WHO Trials Register | Multidisciplinary | Global | Trials register | | Clinical trials register | | **R-NJA** | 84 |  |  |
| International Standard Randomised Controlled Trial Number | ISRCTN | Controlled Trials | Subject-specific | Global | Trials register | | Clinical trials register | | **R-NJA** | 30 |  |  |
| Iranian Registry of Clinical Trials | IRCT |  | Multidisciplinary | Region-specific | Trials register | | Clinical trials register | | **R-NJA** | 1 |  |  |
| Japan Registry of Clinical Trials | jRCT | Japanese Clinical Trials Registry | Multidisciplinary | Region-specific | Trials register | | Clinical trials register | | **R-NJA** | 1 |  |  |
| Japanese University Hospital Medical Information Network | UMIN |  | Multidisciplinary | Region-specific | Trials register | | Clinical trials register | | **R-NJA** | 2 |  |  |
| metaRegister of Controlled Trials | mRCT |  | Multidisciplinary | Global | Trials register | | Clinical trials register | | **R-NJA** | 7 |  |  |
| NCI-Supported Clinical Trial | NTR |  | Subject-specific | Region-specific | Trials register | | Clinical trials register | | **R-NJA** | 1 |  |  |
| Netherlands National Trials Register |  |  | Multidisciplinary | Region-specific | Trials register | | Clinical trials register | | **R-NJA** | 1 |  |  |
| Pharmaceutical Clinical trials register |  |  | Multidisciplinary | Global | Trials register | | Clinical trials register | | **R-NJA** | 5 |  |  |
| Stroke Trials Registry |  |  | Subject-specific | Global | Trials register | | Clinical trials register | | **R-NJA** | 2 |  |  |
| The Brazilian Registry of Clinical Trials | REBEC | Registro Brasileiro de Ensaios Clinicos | Multidisciplinary | Region-specific | Trials register | | Clinical trials register | | **R-NJA** | 3 |  |  |
| The International Prospective Register of Systematic Reviews | PROSPERO |  | Multidisciplinary | Global | Trials register | | Clinical trials register | | **R-NJA** | 8 |  |  |
| The Union Clinical Trials | UKCTG |  | Subject-specific | Global | Trials register | | Clinical trials register | | **R-NJA** | 1 |  |  |
| UK Clinical Trials Gateway |  | NIHR/UK Clinical Trials Network | Multidisciplinary | Region-specific | Trials register | | Clinical trials register | | **R-NJA** | 3 |  |  |
| University/Hospital-Specific Clinical trials register |  | clinicaltrials.org | Subject-specific | Region-specific | Trials register | | Clinical trials register | | **R-NJA** | 4 |  |  |
| Trial Register (not otherwise specified) |  |  | Multidisciplinary | Global | Trials register | | Clinical trials register | | **R-NJA** | 4 |  |  |
| **Cochrane Specialised Registers** |  |  |  | | |  | |  | | |  |  |
| Cochrane Airways Trials Register |  |  | Subject-specific | Global | Cochrane Specialised Register | | Various | | R-MIX | 1 |  |  |
| Cochrane Back and Neck Trials Register |  |  | Subject-specific | Global | Cochrane Specialised Register | | Various | | R-MIX | 1 |  |  |
| Cochrane Colorectal Cancer Group specialised register SR-COLOCA |  |  | Subject-specific | Global | Cochrane Specialised Register | | Various | | R-MIX | 1 |  |  |
| Cochrane Common Mental Disorders Controlled Trials Register |  |  | Subject-specific | Global | Cochrane Specialised Register | | Various | | R-MIX | 1 |  |  |
| Cochrane Cystic Fibrosis and Genetic Disorders Group's Haemoglobinopathies Trials Register |  |  | Subject-specific | Global | Cochrane Specialised Register | | Various | | R-MIX | 1 |  |  |
| Cochrane Cystic Fibrosis and Genetic Disorders Group's Inborn Errors of Metabolism Trial Register |  |  | Subject-specific | Global | Cochrane Specialised Register | | Various | | R-MIX | 2 |  |  |
| Cochrane Dementia and Cognitive Improvement Group's Specialise Register |  |  | Subject-specific | Global | Cochrane Specialised Register | | Various | | R-MIX | 1 |  |  |
| Cochrane Depression Anxiety and Neurosis Group Trials Register |  |  | Subject-specific | Global | Cochrane Specialised Register | | Various | | R-MIX | 2 |  |  |
| Cochrane Developmental, Psychosocial and Learning Problems Specialised Register |  |  | Subject-specific | Global | Cochrane Specialised Register | | Various | | R-MIX | 1 |  |  |
| Cochrane Gynaecology and Fertility Specialised Register of Controlled Trials |  |  | Subject-specific | Global | Cochrane Specialised Register | | Various | | R-MIX | 1 |  |  |
| Cochrane IBD Group Specialised Register |  |  | Subject-specific | Global | Cochrane Specialised Register | | Various | | R-MIX | 3 |  |  |
| Cochrane Incontinence Specialised Register |  |  | Subject Specific | Global | Cochrane Specialised Register | | Various | | R-MIX | 1 |  |  |
| Cochrane Infectious Diseases Group Register |  |  | Subject-specific | Global | Cochrane Specialised Register | | Various | | R-MIX | 2 |  |  |
| Cochrane Injuries Group Specialised Register |  |  | Subject-specific | Global | Cochrane Specialised Register | | Various | | R-MIX | 3 |  |  |
| Cochrane Kidney and Transplant Register |  |  | Subject-specific | Global | Cochrane Specialised Register | | Various | | R-MIX | 2 |  |  |
| Cochrane Methodology Registry |  |  | Subject-specific | Global | Cochrane Specialised Register | | Various | | R-MIX | 1 |  |  |
| Cochrane Multiple Sclerosis and Rare Diseases Specialised Register |  |  | Subject Specific | Global | Cochrane Specialised Register | | Various | | R-MIX | 1 |  |  |
| Cochrane Neuromuscular Specialised Register |  |  | Subject-specific | Global | Cochrane Specialised Register | | Various | | R-MIX | 2 |  |  |
| Cochrane Oral Health Group Specialised Register |  |  | Subject-specific | Global | Cochrane Specialised Register | | Various | | R-MIX | 6 |  |  |
| Cochrane Pregnancy and Childbirth Group Trials Register |  |  | Subject-specific | Global | Cochrane Specialised Register | | Various | | R-MIX | 4 |  |  |
| Cochrane Schizophrenia Group's Study‐Based Register of Trials |  |  | Subject-specific | Global | Cochrane Specialised Register | | Various | | R-MIX | 3 |  |  |
| Cochrane Stroke Group Register |  |  | Subject-specific | Global | Cochrane Specialised Register | | Various | | R-MIX | 2 |  |  |
| Cochrane Wounds Specialised Register |  |  | Subject-specific | Global | Cochrane Specialised Register | | Various | | R-MIX | 1 |  |  |
| **Evidence-based medicine databases** |  |  |  | | |  | |  | | |  |  |
| American College of Physicians Journal Club | ACP Journal Club |  | Multidisciplinary | Global | EBM Database | | EBM compilation | | R-MIX | 2 |  |  |
| Bandolier |  |  | Multidisciplinary | Global | EBM Database | | EBM compilation | | R-MIX | 1 |  |  |
| Best Bets |  |  | Multidisciplinary | Global | EBM Database | | EBM compilation | | R-MIX | 1 |  |  |
| Best Practice BMJ | BMJ |  | Multidisciplinary | Global | EBM Database | | EBM compilation | | R-MIX | 3 |  |  |
| The Campbell Collaboration |  |  | Subject-specific | Global | EBM Database | | EBM compilation | | R-MIX | 1 |  |  |
| Cochrane Library |  |  | Multidisciplinary | Global | Bibliographic database | | Journals (systematic reviews), journals, conference proceedings, EBM compilations, regulatory data, trial | | R-MIX | 311 |  |  |
| ** Cochrane Clinical Answers* | CCA |  | Multidisciplinary | Global | EBM Database | | EBM compilation | | R-MIX | 0 |  |  |
| Centre for Reviews and Dissemination database (Health Technology Assessments and the Database of Abstracts and Reviews of Effects) |  |  | Multidisciplinary | Region-specific | EBM Database | | EBM compilation | | R-MIX | 1 |  |  |
| ** Database of Abstracts and Reviews of Effects* | DARE |  | Multidisciplinary | Region-specific | Bibliographic database | | Bibliographic database | | R-MIX | 18 |  |  |
| Clinical Evidence |  |  | Multidisciplinary | Region-specific | EBM Database | | EBM compilation | | R-MIX | 1 |  |  |
| Dynamed |  |  | Multidisciplinary | Global | EBM Database | | EBM compilation | | R-MIX | 1 |  |  |
| Epistemonikos |  |  | Multidisciplinary | Global | EBM Database | | EBM compilation | | R-MIX | 7 |  |  |
| Evidence-Based Medicine Reviews | EBMR |  | Multidisciplinary | Global | EBM Database | | EBM compilation | | R-MIX | 1 |  |  |
| Physicians Data Query | PDQ |  | Multidisciplinary | Global | EBM Database | | EBM compilation | | R-MIX | 1 |  |  |
| UpToDate |  |  | Multidisciplinary | Global | EBM Database | | EBM compilation | | R-MIX | 3 |  |  |
| Virtual Health Library | VHL |  | Multidisciplinary | Region-specific | Bibliographic Database | | Journals, theses, monographs, books, conference proceedings, reports, government publications and publications by international organisations | | R-MIX | 12 |  |  |
| ** Formative Second Opinion* | SOF |  | Multidisciplinary | Global | EBM Bulletin | | EBM compilation | | R-MIX | 0 |  |  |
| **Government agency databases** |  |  |  | | |  | |  | | |  |  |
| Agency for Healthcare Research and Quality | AHRQ |  | Multidisciplinary | Region-specific | Bibliographic database | | Government documents | | **R-NJA** | 4 |  |  |
| Association of the Scientific Medical Societies in Germany | AWMF | Arbeitsgemeinschaft der Wissenschaftlichen Medizinischen Fachgesellschaften | Multidisciplinary | Region-specific | Bibliographic Database | | Guidelines | | **R-NJA** | 2 |  |  |
| Canadian Agency for Drugs and Technologies in Health | CADTH |  | Multidisciplinary | Region-specific | Government Agency Database | | Reports, HTA assessments | | **R-NJA** | 2 |  |  |
| Center for Drug Evaluation and Research | CDER | Drugs@FDA.gov | Multidisciplinary | Region-specific | Bibliographic Database | | Regulatory data | | **R-NJA** | 1 |  |  |
| Centre for Reviews and Dissemination database (Health Technology Assessments and the Database of Abstracts and Reviews of Effects) |  |  | Multidisciplinary | Region-specific | Bibliographic database | | Bibliographic database | | R-MIX | 1 |  |  |
| ** Database of Abstracts and Reviews of Effects* |  |  | Multidisciplinary | Region-specific | Bibliographic database | | Bibliographic database | | R-MIX | 18 |  |  |
| ** Health Technology Assessments* |  |  | Multidisciplinary | Region-specific | Bibliographic database | | Health technology assessments | | **R-NJA** | 4 |  |  |
| Dutch Guidelines |  | Oncoline.nl | Subject-specific | Region-specific | Bibliographic Database | | Guidelines | | **R-NJA** | 1 |  |  |
| FDA |  |  | Multidisciplinary | Region-specific | Bibliographic database | | Guidance and regulatory data | | **R-NJA** | 1 |  |  |
| German Agency for Quality in Medicine | ÄZQ | Ärztliches Zentrum für Qualität in der Medizin | Multidisciplinary | Region-specific | Government Agency Database | | Guidelines | | **R-NJA** | 1 |  |  |
| Medicines Commision of the German Medical Association | AkdÄ | Arzneimittelkommission der deutschen Ärzteschaft | Multidisciplinary | Region-specific | Bibliographic Database | | Guidelines | | **R-NJA** | 1 |  |  |
| National Institute for Health and Care Excellence | NICE | NHS Evidence | Multidisciplinary | Region-specific | Bibliographic Database | | Guidelines | | **R-NJA** | 7 |  |  |
| NHS Economic Evaluation Database | NHSEED |  | Multidisciplinary | Region-specific | Bibliographic Database | | HTAs | | **R-NJA** | 12 |  |  |
| Scottish Intercollegiate Guidelines Network | SIGN |  | Multidisciplinary | Region-specific | Bibliographic Database | | Guidelines | | **R-NJA** | 1 |  |  |
| The Scottish Medicines Consortium | SMC |  | Multidisciplinary | Region-specific | Bibliographic Database | | Guidance and regulatory data | | **R-NJA** | 1 |  |  |
| WHO (not otherwise specified) | WHOLIS |  | Multidisciplinary | Global | Bibliographic Database | | Journals, guidelines, evidence-based bulletins, datasets, conference proceedings, bibliographic registers | | R-MIX | 5 |  |  |
| **Search engines** |  |  |  |  |  | |  | |  |  |  |  |
| Google |  |  | Multidisciplinary | Global | Search Engine | | World wide web | | R-MIX | 17 |  |  |
| Google Scholar |  |  | Multidisciplinary | Global | Search Engine | | World wide web | | R-MIX | 196 |  |  |
| LIVIVO |  |  | Multidisciplinary | Global | Search Engine | | World wide web | | R-MIX | 3 |  |  |
| MedNAR |  |  | Multidisciplinary | Global | Search Engine | | World wide web | | R-MIX | 4 |  |  |
| Translating Research Into Medical Practice Medical Database | Trip Medical Database |  | Multidisciplinary | Global | Search Engine | | World wide web | | R-MIX | 8 |  |  |
| WorldWideScience.org |  |  | Multidisciplinary | Global | Search Engine | | World wide web | | R-MIX | 1 |  |  |
| **Other search facilities** |  |  |  |  |  | |  | |  |  |  |  |
| Search Book |  |  | Multidisciplinary | Global | Search | | Books | | R-MIX | 8 |  |  |
| Search Bulletins |  |  | Multidisciplinary | Global | Search | | Bulletins | | **R-NJA** | 1 |  |  |
| Search Conference Proceedings |  |  | Multidisciplinary | Global | Search | | Conference proceedings | | **R-NJA** | 68 |  |  |
| Search Editorials |  |  | Multidisciplinary | Global | Search | | Editorials | | **R-NJA** | 1 |  |  |
| Search Governance Documents |  |  | Multidisciplinary | Global | Search | | Governance documents | | **R-NJA** | 1 |  |  |
| Search Government Documents |  |  | Multidisciplinary | Global | Search | | Government documents | | **R-NJA** | 1 |  |  |
| Search Guidelines |  |  | Multidisciplinary | Global | Search | | Guidelines | | **R-NJA** | 2 |  |  |
| Search Health Technology Assessments |  |  | Multidisciplinary | Global | Search | | Health technology assessments | | **R-NJA** | 1 |  |  |
| Search Internal Reports |  |  | Multidisciplinary | Global | Search | | Internal reports | | **R-NJA** | 5 |  |  |
| Search Journals |  |  | Multidisciplinary | Global | Search | | Journals | | R-JA | 46 |  |  |
| “Search” (not otherwise specified) |  |  | Multidisciplinary | Global | Search | | (not otherwise specified) | | R-MIX | 3 |  |  |
| Search Theses |  |  | Multidisciplinary | Global | Search | | Theses | | **R-NJA** | 34 |  |  |
| Search Websites |  |  | Multidisciplinary | Global | Search | | Websites | | **R-NJA** | 4 |  |  |
| **Miscellaneous** |  |  |  |  |  | |  | |  |  |  |  |
| Author's Personal Collection |  |  | Multidisciplinary | Global | Personal Collection | | Any kind of scientific work | | R-MIX | 9 |  |  |
| Compendium of Neuropsychological tests |  |  | Subject-specific | Global | Book | | Book | | **R-NJA** | 1 |  |  |
| Contact Experts |  |  | Multidisciplinary | Global | Contact | | Any kind of scientific work | | **R-NJA** | 78 |  |  |
| Email Alert |  |  | Multidisciplinary | Global | Email Alert | | Email alert | | R-MIX | 1 |  |  |
| Research Gate |  |  | Multidisciplinary | Global | Social Media | | Social media | | R-MIX | 5 |  |  |
| Social Media (not otherwise specified) |  |  | Multidisciplinary | Global | Social Media | | Social media | | R-MIX | 1 |  |  |
| **Unclassifiable** |  |  |  |  |  | |  | |  |  |  |  |
| Drug Interaction Checker |  |  | Multidisciplinary | Global | N/A | | N/A | | N/A | 2 |  |  |
| Ensembl (Genetic Database) |  |  | Multidisciplinary | Global | N/A | | N/A | | N/A | 1 |  |  |
| Genetic Database |  |  | Multidisciplinary | Global | N/A | | N/A | | N/A | 1 |  |  |
| Search Tools |  |  | Multidisciplinary | Global | N/A | | N/A | | N/A | 1 |  |  |
| **Irretrievable resources** |  |  |  |  |  | |  | |  |  |  |  |
| BARAKAT |  |  |  |  |  | |  | |  | 1 |  |  |
| Chocane |  |  |  |  |  | |  | |  | 1 |  |  |
| FIS |  |  |  |  |  | |  | |  | 1 |  |  |
| Grey Literature Support |  |  |  |  |  | |  | |  | 1 |  |  |
| Health Foundation, Evidence Based Midwifery and Analysis & Policy Observation |  |  |  |  |  | |  | |  | 1 |  |  |
| Institute of Epidemiology and Tropical Neurology databases |  |  |  |  |  | |  | |  | 1 |  |  |
| MedElPORTAL |  |  |  |  |  | |  | |  | 1 |  |  |
| Medic |  |  |  |  |  | |  | |  | 1 |  |  |
| National Dental Trials Registry |  |  |  |  |  | |  | |  | 1 |  |  |
| NDSL |  |  |  |  |  | |  | |  | 2 |  |  |
| Santé Com |  |  |  |  |  | |  | |  | 1 |  |  |
| Science Online |  |  |  |  |  | |  | |  | 1 |  |  |
| Spanish Medical Index |  |  |  |  |  | |  | |  | 1 |  |  |
| SPECTR |  |  |  |  |  | |  | |  | 6 |  |  |
| Stroke Trials Registry |  |  |  |  |  | |  | |  | 2 |  |  |
| Tobacco Addiction Register of Clinical Trials |  |  |  |  |  | |  | |  | 1 |  |  |

N/A = Not applicable

*Asterisked and italicised entries are sub-databases of the wider source listed above.

†Number of reviews included in our empirical study that referenced searching of this source.

**Appendix C: Categorisation of each journal* (n=649) by subject, according to ASCJ Coding and clinical judgement**

(1) Biomedical, Biostatistics & Laboratory Sciences (n=80); (2) Clinical & Procedural Medicine (n=168); (3) Internal Medicine & Chronic Disease (n=145); (4) Mental, Neurological & Behavioural Health (n=88); (5) Pharmacology & Therapeutics (n=37); (6) Public Health & Health Systems (n=88); (7) Reproductive, Maternal & Child Health (n=43)

| **Biomedical, Biostatistics & Laboratory (n=80)** | **Clinical & Procedural Medicine (n=168)** | **Internal Medicine & Chronic Disease (n=145)** | **Mental, Neurological & Behavioural Health (n=88)** |
| --- | --- | --- | --- |
| Cureus | BMC Neurology | European Respiratory Review | Clinical Neuropharmacology 88 |
| Frontiers in Physiology | Urologia Internationalis | BMJ Open | Neurosurgical Review |
| PLOS ONE | International Journal of Oral and Maxillofacial Surgery | American Journal of Cardiovascular Drugs | Developmental Neurorehabilitation |
| Animals | Journal of Science and Medicine in Sport | Nutrition, Metabolism and Cardiovascular Diseases | BMC Psychiatry |
| Sensors | Dental Traumatology | Research Quarterly for Exercise and Sport | Clinical Psychology Review |
| BMC Complementary and Alternative Medicine | Community Dental Health | Medicine (United States) | Australian and New Zealand Journal of Psychiatry |
| Systematic Reviews | Dentomaxillofacial Radiology | Archives of Gerontology and Geriatrics | Asian Journal of Psychiatry |
| American Journal of Infection Control | Experimental and Clinical Transplantation | Dermatology | Academic Psychiatry |
| Biomedical Research and Therapy | Frontiers in Oncology | Gerontology and Geriatric Medicine | The Lancet Psychiatry |
| Cytokine | Physical Therapy in Sport | Current Diabetes Reviews | Psychiatry Research |
| Journal of Microbiology, Immunology and Infection | Journal of Orthopaedic and Sports Physical Therapy | Tunisie Medicale | Frontiers in Psychiatry |
| Analyst | BMC Musculoskeletal Disorders | Journal of Cardiac Failure | Archives of Physical Medicine and Rehabilitation |
| Molecular Psychiatry | European Journal of Surgical Oncology | eBioMedicine | Sleep Health |
| ACS Chemical Neuroscience | Orthopaedics and Traumatology: Surgery and Research | Diabetes Research and Clinical Practice | Psychiatric Quarterly |
| RMD Open | Australian Critical Care | HPB | Brain and Behavior |
| American Journal of Clinical Pathology | Techniques in Coloproctology | Metabolism: Clinical and Experimental | Journal of Affective Disorders |
| Evidence-based Complementary and Alternative Medicine | Sports Medicine | American Journal of Clinical Dermatology | Journal of Child Psychology and Psychiatry and Allied Disciplines |
| Biochimie | Clinical Implant Dentistry and Related Research | Expert Review of Gastroenterology and Hepatology | Frontiers in Psychology |
| Clinical Microbiology and Infection | Surgical Oncology | Journal of Thoracic Disease | Stroke |
| Transfusion | Neuroscience and Biobehavioral Reviews | American Journal of Cardiology | Journal of Neuroinflammation |
| Journal of Acquired Immune Deficiency Syndromes | Resuscitation | Andrology | Epilepsy and Behavior |
| Journal of Manipulative and Physiological Therapeutics | Frontiers in Neurology | Journal of Clinical Medicine | Acta Neurochirurgica |
| Journal of Immunology Research | Journal of Evidence-Based Dental Practice | Saudi Journal of Gastroenterology | Neuromodulation |
| Current Microbiology | Clinical Nutrition | International Journal of Colorectal Disease | Journal of Substance Abuse Treatment |
| Cell Proliferation | Journal of the American College of Surgeons | JAMA Network Open | Neurological Sciences |
| Disease Markers | Clinical Neurology and Neurosurgery | Kidney and Blood Pressure Research | Drug and Alcohol Review |
| Quintessence International | Journal of Vascular Surgery: Venous and Lymphatic Disorders | Archives of Medical Science | Journal of Psychosomatic Research |
| Journal of Cellular Physiology | Archives of Oral Biology | Journal of Gastroenterology and Hepatology (Australia) | Aging and Disease |
| American Journal of Chinese Medicine | Journal of Oral Biology and Craniofacial Research | BMC Gastroenterology | European Journal of Psychotraumatology |
| Brazilian Journal of Microbiology | Operative Dentistry | Materials Science and Engineering C | American Journal of Physical Medicine and Rehabilitation |
| JBI Database of Systematic Reviews and Implementation Reports | Anaesthesiology Intensive Therapy | Metabolomics | Journal of ECT |
| Bioscience Reports | Archives of Bone and Joint Surgery | Journal of Thrombosis and Thrombolysis | Multiple Sclerosis and Related Disorders |
| Gene | Anesthesia and Analgesia | Journal of Thoracic and Cardiovascular Surgery | General Hospital Psychiatry |
| Journal of Travel Medicine | BMC Cancer | Diabetes/Metabolism Research and Reviews | Neuropsychology Review |
| Microbial Pathogenesis | Breast Cancer Research and Treatment | Diabetes and Metabolism | Depression and Anxiety |
| Transfusion Medicine Reviews | Pathology and Oncology Research | Respiration | Journal of the American Academy of Child and Adolescent Psychiatry |
| Journal of Education and Health Promotion | Neurology: Clinical Practice | Heart Failure Reviews | Frontiers in Aging Neuroscience |
| International Braz J Urol | Critical Reviews in Oncology/Hematology | Medical Archives | Journal of Molecular Neuroscience |
| Journal of Steroid Biochemistry and Molecular Biology | Dental Journal | Angiology | Sleep Medicine Reviews |
| Pathology Research and Practice | Journal of Prosthetic Dentistry | Journal of Cardiovascular Medicine | Journal of Psychiatric Research |
| Endocrine Pathology | International Orthopaedics | Obesity Research and Clinical Practice | Schizophrenia Research |
| Prostaglandins Leukotrienes and Essential Fatty Acids | Surgical Endoscopy | Aging Clinical and Experimental Research | Epilepsia |
| Forensic Science International | British Journal of Surgery | Diabetic Medicine | Journal of Stroke and Cerebrovascular Diseases |
| Angle Orthodontist | Orthopaedic Surgery | Journal of Interventional Cardiology | Science of the Total Environment |
| Wellcome Open Research | Journal of Plastic, Reconstructive and Aesthetic Surgery | Journal of Diabetes Research | Translational Stroke Research |
| Materials | Asian Pacific Journal of Cancer Prevention | Diabetologia | Frontiers in Neuroscience |
| Cell Stress and Chaperones | Journal of the American Dental Association | BMC Geriatrics | Brain Sciences |
| Frontiers in Bioengineering and Biotechnology | Plastic and Reconstructive Surgery | Clinics | Journal of Addictions Nursing |
| Canadian Family Physician | Australian Dental Journal | Osteoarthritis and Cartilage | Sleep Medicine Research |
| Neurobiology of Stress | International Journal of Surgery | Indian Heart Journal | World Neurosurgery |
| Reviews in Medical Virology | Injury | Lipids in Health and Disease | Journal of Neuroradiology |
| Journal of the European Academy of Dermatology and Venereology | Cancer Epidemiology Biomarkers and Prevention | Biomedical Research and Therapy | Comprehensive Psychiatry |
| European Journal of Physical Medicine and Rehabilitation | Surgical Neurology International | Current Cardiology Reviews | Journal of Neurological Sciences |
| Pharmacology Biochemistry and Behavior | Ocular Oncology and Pathology | Hormone Molecular Biology and Clinical Investigation | Clinical Neurophysiology |
| Free Radical Research | Journal of Oral Rehabilitation | Gastroenterology and Endoscopy | Pain Research and Management |
| Physiological Reports | World Journal of Urology | Journal of Translational Medicine | Translational Psychiatry |
| Clinical Immunology | Clinical Biomechanics | Journal of Invasive Cardiology | Global Spine Journal |
| Genetic Testing and Molecular Biomarkers | Interactive Cardiovascular and Thoracic Surgery | International Journal of Angiology | Seizure |
| International Archives of Allergy and Applied Immunology | Eye (Basingstoke) | Frontiers in Endocrinology | Journal of Wound Care |
| Clinical and Molecular Hepatology | Contemporary Clinical Dentistry | Annals of Gastroenterological Surgery | Schizophrenia Research: Cognition |
| Annals of Hematology | Journal of Cancer | European Journal of Dermatology | Spine Journal |
| International Journal of Hyperthermia | European Journal of Paediatric Neurology | Journal of Musculoskeletal Neuronal Interactions | NeuroImage: Clinical |
| IUBMB Life | AORN Journal | BMC Nephrology | Spinal Cord |
| Journal of Clinical Periodontology | Pain Physician | Danish Medical Journal | Sleep and Breathing |
| International Journal of Antimicrobial Agents | Journal of Orthopaedic Surgery and Research | Journal of the American Pharmaceutical Association | Ageing Research Reviews |
| Histopathology | Critical Care | Primary Care Diabetes | Substance Abuse: Treatment, Prevention, and Policy |
| Clinica Chimica Acta | American Journal of Surgery | International Journal of Dermatology | Journal of Child and Adolescent Mental Health |
| Journal of Inherited Metabolic Disease | European Radiology | Clinical Obesity | Addiction |
| Molecular Biology Reports | Head and Neck | Journal of Clinical Hypertension | American Journal of Neuroradiology |
| Cellular and Molecular Life Sciences | Brazilian Oral Research | International Journal of Clinical Practice | Journal of Neurogastroenterology and Motility |
| Cells | European Journal of Pain | Medical Problems of Performing Artists | Journal of NeuroInterventional Surgery |
| Vaccine | International Journal of Oral Implantology | Colorectal Disease | Parkinsonism and Related Disorders |
| Journal of Alternative and Complementary Medicine | European Journal of Trauma and Emergency Surgery | Indian Journal of Endocrinology and Metabolism | Spine |
| Influenza and other Respiratory Viruses | European Spine Journal | Alimentary Pharmacology and Therapeutics | Scandinavian Journal of Psychology |
| Complementary Therapies in Clinical Practice | Acta Oncologica | Frontiers in Cardiovascular Medicine | Otology and Neurotology |
| Immunological Investigations | Laryngoscope | Endoscopy International Open | Movement Disorders |
| Journal of Clinical Pathology | Oral Health and Preventive Dentistry | Diseases of the Colon and Rectum | Psychoneuroendocrinology |
| Histology and Histopathology | Hernia | Hemodialysis International | Journal of Autism and Developmental Disorders |
| Oxidative Medicine and Cellular Longevity | Pain Practice | Mayo Clinic Proceedings | Appetite |
| Sexually Transmitted Infections | British Journal of Oral and Maxillofacial Surgery | Heart | Journal of Forensic Nursing |
|  | Oral Oncology | Reviews in Endocrine and Metabolic Disorders | Body Image |
|  | Radiotherapy and Oncology | Medicina (Lithuania) | Psychology, Health and Medicine |
|  | European Journal of Orthopaedic Surgery and Traumatology | Patient Education and Counseling | Journal of Consulting and Clinical Psychology |
|  | Journal of Behavioral Addictions | European Journal of Vascular and Endovascular Surgery | Headache |
|  | CiOS Clinics in Orthopedic Surgery | Military Medical Research | Neural Plasticity |
|  | World Journal of Surgery | Clinical Interventions in Aging | Journal of Intellectual Disability Research |
|  | Critical Care Medicine | PLOS Medicine | Journal of Applied Research in Intellectual Disabilities |
|  | Japanese Journal of Clinical Oncology | Diabetes, Metabolic Syndrome and Obesity | Clinical Psychology and Psychotherapy |
|  | World Journal of Surgical Oncology | Cardiology and Therapy |  |
|  | Journal of Neurology | Hormones | **Reproductive, Maternal & Child Health** |
|  | Journal of Cardiac Surgery | Journal of Women's Health | Nurse Education in Practice |
|  | Annals of Physical and Rehabilitation Medicine | Acta Paediatrica Belgica | International Journal of Nursing Sciences |
|  | Brazilian Journal of Otorhinolaryngology | Journal of Multidisciplinary Healthcare | Acta Paediatrica Belgica |
|  | Clinical Oral Investigations | F1000Research | European Journal of Obstetrics and Gynecology and Reproductive Biology |
|  | Journal of Contemporary Dental Practice | Journal of Gastrointestinal and Liver Diseases | International Journal of Pediatric Otorhinolaryngology |
|  | BMJ Open Sport and Exercise Medicine | Osteoporosis International | Nurse Education Today |
|  | International Journal of Gynecological Cancer | Medical Science Monitor | Journal of Advanced Nursing |
|  | Journal of Adhesive Dentistry | EuroIntervention | Reproductive BioMedicine Online |
|  | Orthopaedic Journal of Sports Medicine | Investigacion y Educacion en Enfermeria | Worldviews on Evidence-Based Nursing |
|  | BMJ Supportive and Palliative Care | Pediatric Pulmonology | Asian Nursing Research |
|  | European Archives of Oto-Rhino-Laryngology | Acta Clinica Belgica: International Journal of Clinical and Laboratory Medicine | Obstetrical and Gynecological Survey |
|  | Renal Failure | BMC Palliative Care | Pediatrics |
|  | Arthroscopy - Journal of Arthroscopic and Related Surgery | Digestive and Liver Disease | Taiwanese Journal of Obstetrics and Gynecology |
|  | British Journal of Anaesthesia | Sleep Medicine | Journal of Minimally Invasive Gynecology |
|  | Anticancer Research | Annals of Internal Medicine | Iranian Journal of Nursing and Midwifery Research |
|  | Archives of Osteoporosis | Nutrition Research | Revista Paulista de Pediatria |
|  | ANZ Journal of Surgery | BMC Endocrine Disorders | Complementary Therapies in Medicine |
|  | Supportive Care in Cancer | World Journal of Gastroenterology | Ultrasound in Obstetrics and Gynecology |
|  | Clinical and Experimental Metastasis | Open Access Rheumatology: Research and Reviews | Maturitas |
|  | Burns | Netherlands Heart Journal | Critical Care Nursing Quarterly |
|  | Cancers | BMJ | Journal of Clinical Nursing |
|  | Foot and Ankle Surgery | Internal Medicine Journal | BMC Pregnancy and Childbirth |
|  | International Urology and Nephrology | Journal of Cardiovascular Pharmacology | Archives of Disease in Childhood: Fetal and Neonatal Edition |
|  | Oral and Maxillofacial Surgery | JAMA Internal Medicine | Archives of Women's Mental Health |
|  | Neurology | Archives of Iranian Medicine | Human Reproduction Update |
|  | International Journal of Dental Hygiene | Indian Journal of Gastroenterology | Obstetrics and Gynecology |
|  | Perioperative Medizin | Journal of Nephrology | Journal of Paediatrics and Child Health |
|  | Integrative Cancer Therapies | Annals of Hepatology | International Journal of Gynecology and Obstetrics |
|  | Psycho-Oncology | Revista Espanola de Enfermedades Digestivas | Revista Brasileira de Enfermagem |
|  | American Journal of Emergency Medicine | Circulation: Cardiovascular Imaging | BMC Pediatrics |
|  | Oral Diseases | Thyroid | Midwifery |
|  | International Journal of Paediatric Dentistry | British Journal of Dermatology | Frontiers in Pediatrics |
|  | Journal of Cardiothoracic Surgery | Seminars in Arthritis and Rheumatism | American Journal of Reproductive Immunology |
|  | Journal of the Korean Association of Oral and Maxillofacial Surgeons | Amyloid | Pediatric Research |
|  | Cancer Cell International | BMC Cardiovascular Disorders | Prenatal Diagnosis |
|  | Journal of Cancer Survivorship | Respiratory Research | Archives of Gynecology and Obstetrics |
|  | Neurourology and Urodynamics | American Journal of Gastroenterology | Gynecologic and Obstetric Investigation |
|  | Critical Care and Resuscitation | Clinical Toxicology | BMJ Sexual and Reproductive Health |
|  | European Journal of Nuclear Medicine and Molecular Imaging | Gastroenterology | International Journal of Palliative Nursing |
|  | Cancer Imaging | American Heart Journal | BMC Women's Health |
|  | Transplantation Proceedings | Biological Trace Element Research | Pediatric Physical Therapy |
|  | Journal of Shoulder and Elbow Surgery | Cardiovascular Engineering and Technology | Clinical Child and Family Psychology Review |
|  | Community Dentistry and Oral Epidemiology | Journal of Cardiovascular Translational Research | Pediatric Rheumatology |
|  | Journal of Sports Science and Medicine | CardioRenal Medicine |  |
|  | Breast Cancer | Cardiovascular Toxicology |  |
|  | Bone and Joint Journal | Acta Diabetologica |  |
|  | Emergency Medicine Journal | Eastern Mediterranean Health Journal |  |
|  | Journal of Oral and Maxillofacial Surgery | The Lancet Global Health |  |
|  | European Journal of Radiology | Diabetology and Metabolic Syndrome |  |
|  | Acta Radiologica | Diagnostics |  |
|  | Neurology India | Psychogeriatrics |  |
|  | Cancer and Metastasis Reviews | Australasian Journal of Dermatology |  |
|  | Journal of Cachexia, Sarcopenia and Muscle | Journal of Clinical Densitometry |  |
|  | Annals of Nuclear Medicine | Canadian Journal of Cardiology |  |
|  | European Urology Focus | Journal of Cardiovascular and Thoracic Research |  |
|  | Future Oncology |  |  |
|  | BMC Urology |  |  |
|  | British Journal of Urology |  |  |
|  | American Journal of Roentgenology |  |  |
|  | Journal for ImmunoTherapy of Cancer |  |  |
|  | Transplantation Reviews |  |  |
|  | Journal of Vascular Nursing |  |  |
|  | British Journal of Sports Medicine |  |  |
|  | BMC Anesthesiology |  |  |
|  | Journal of Audiology and Otology |  |  |
|  | BMC Emergency Medicine |  |  |
|  | European Journal of Oncology Nursing |  |  |
|  | International Journal of Behavioral Nutrition and Physical Activity |  |  |
|  | Advances in Wound Care |  |  |
|  | Photobiomodulation, Photomedicine, and Laser Surgery |  |  |
|  | Musculoskeletal Care |  |  |
|  | Medical Ultrasonography |  |  |
|  | Brazilian Dental Journal |  |  |
|  | Dental Materials |  |  |
|  | Sports Medicine - Open |  |  |
|  | Prehospital and Disaster Medicine |  |  |
|  | Acta Anaesthesiologica Scandinavica |  |  |
|  | Otolaryngology - Head and Neck Surgery |  |  |
|  |  |  |  |

| **Pharmacology & Therapeutics (n=37)** | **Public Health & Health Systems (n=88)** | **Reproductive, Maternal & Child Health (n=43)** |
| --- | --- | --- |
| Frontiers in Pharmacology (37) | Nurse Education in Practice | Nurse Education in Practice |
| Inflammation Research | International Journal of Nursing Sciences | International Journal of Nursing Sciences |
| Orphanet Journal of Rare Diseases | Acta Paediatrica Belgica | Acta Paediatrica Belgica |
| DARU, Journal of Pharmaceutical Sciences | European Journal of Obstetrics and Gynecology and Reproductive Biology | European Journal of Obstetrics and Gynecology and Reproductive Biology |
| Annals of Pharmacotherapy | International Journal of Pediatric Otorhinolaryngology | International Journal of Pediatric Otorhinolaryngology |
| Phytotherapy Research | Nurse Education Today | Nurse Education Today |
| Pharmacy Education | Journal of Advanced Nursing | Journal of Advanced Nursing |
| Cardiovascular Drugs and Therapy | Reproductive BioMedicine Online | Reproductive BioMedicine Online |
| Pharmazie | Worldviews on Evidence-Based Nursing | Worldviews on Evidence-Based Nursing |
| European Journal of Clinical Pharmacology | Asian Nursing Research | Asian Nursing Research |
| Rheumatology | Obstetrical and Gynecological Survey | Obstetrical and Gynecological Survey |
| Pharmacological Research | Pediatrics | Pediatrics |
| Expert Review of Clinical Pharmacology | Taiwanese Journal of Obstetrics and Gynecology | Taiwanese Journal of Obstetrics and Gynecology |
| Cardiovascular Therapeutics | Journal of Minimally Invasive Gynecology | Journal of Minimally Invasive Gynecology |
| British Journal of Clinical Pharmacology | Iranian Journal of Nursing and Midwifery Research | Iranian Journal of Nursing and Midwifery Research |
| BMC Pharmacology and Toxicology | Revista Paulista de Pediatria | Revista Paulista de Pediatria |
| Currents in Pharmacy Teaching and Learning | Complementary Therapies in Medicine | Complementary Therapies in Medicine |
| Life Sciences | Ultrasound in Obstetrics and Gynecology | Ultrasound in Obstetrics and Gynecology |
| Chinese Medicine (United Kingdom) | Maturitas | Maturitas |
| Drug Design, Development and Therapy | Critical Care Nursing Quarterly | Critical Care Nursing Quarterly |
| Vascular Health and Risk Management | Journal of Clinical Nursing | Journal of Clinical Nursing |
| Photodiagnosis and Photodynamic Therapy | BMC Pregnancy and Childbirth | BMC Pregnancy and Childbirth |
| Toxins | Archives of Disease in Childhood: Fetal and Neonatal Edition | Archives of Disease in Childhood: Fetal and Neonatal Edition |
| Archives of Physical Medicine and Rehabilitation | Archives of Women's Mental Health | Archives of Women's Mental Health |
| Clinical Rehabilitation | Human Reproduction Update | Human Reproduction Update |
| Chiropractic and Manual Therapies | Obstetrics and Gynecology | Obstetrics and Gynecology |
| Journal of Clinical Psychopharmacology | Journal of Paediatrics and Child Health | Journal of Paediatrics and Child Health |
| Planta Medica | International Journal of Gynecology and Obstetrics | International Journal of Gynecology and Obstetrics |
| European Review for Medical and Pharmacological Sciences | Revista Brasileira de Enfermagem | Revista Brasileira de Enfermagem |
| Clinical Pharmacokinetics | BMC Pediatrics | BMC Pediatrics |
| Therapeutic Advances in Respiratory Disease | Midwifery | Midwifery |
| Dose-Response | Frontiers in Pediatrics | Frontiers in Pediatrics |
| Musculoskeletal Science and Practice | American Journal of Reproductive Immunology | American Journal of Reproductive Immunology |
| Drug and Alcohol Dependence | Pediatric Research | Pediatric Research |
| International Journal of Biometeorology | Prenatal Diagnosis | Prenatal Diagnosis |
| Journal of Orthopaedic and Sports Physical Therapy | Archives of Gynecology and Obstetrics | Archives of Gynecology and Obstetrics |
| Research in Social and Administrative Pharmacy | Gynecologic and Obstetric Investigation | Gynecologic and Obstetric Investigation |
|  | BMJ Sexual and Reproductive Health | BMJ Sexual and Reproductive Health |
|  | International Journal of Palliative Nursing | International Journal of Palliative Nursing |
|  | BMC Women's Health | BMC Women's Health |
|  | Pediatric Physical Therapy | Pediatric Physical Therapy |
|  | Clinical Child and Family Psychology Review | Clinical Child and Family Psychology Review |
|  | Pediatric Rheumatology | Pediatric Rheumatology |
| *Duplicate journals removed |  |  |
