# Supplementary material for: How comprehensive is comprehensive? Prevalence of searches beyond peer-reviewed journal articles and bibliographic sources: a cross-sectional study of systematic reviews
Source: Res Synth Methods. 2026 May 5;17(6):1210–20. doi: 10.1017/rsm.2026.10086 (PMC13732357; doi:10.1017/rsm.2026.10086)
Supplement: Supplementary file 2 [file S1759287926100866sup002.docx]

Supplemental Figure 1a: Proportions of each subcategory of reviews considered to have performed a search for non-journal materials: biomedical, statistics & lab (n=165), clinical & procedural medicine (n=317), internal medicine & chronic disease (n=267), mental, neurological & behavioural (n=142), pharmacology & therapeutics (n=61), public health & health systems (n=158) and reproductive, maternal & child (n=86)

Supplemental Figure 1b: Proportions of each subcategory of reviews considered to have potentially encountered non-journal materials: biomedical, statistics & lab (n=165), clinical & procedural medicine (n=317), internal medicine & chronic disease (n=267), mental, neurological & behavioural (n=142), pharmacology & therapeutics (n=61), public health & health systems (n=158) and reproductive, maternal & child (n=86)
